# Multidimensional Widefield Infrared-Encoded Spontaneous Emission Microscopy: Distinguishing Chromophores by Ultrashort Infrared Pulses

**DOI:** 10.1021/jacs.3c07251

**Published:** 2023-12-12

**Authors:** Chang Yan, Chenglai Wang, Jackson C. Wagner, Jianyu Ren, Carlynda Lee, Yuhao Wan, Shizhen E. Wang, Wei Xiong

**Affiliations:** † Department of Chemistry and Biochemistry, University of California San Diego, La Jolla, California 92093, United States; ‡ Center for Ultrafast Science and Technology, School of Chemistry and Chemical Engineering, Shanghai Jiao Tong University, Shanghai 200240, China; § Zhangjiang Institute for Advanced Study, Shanghai Jiao Tong University, Shanghai 200240, China; ∥ Department of Pathology, University of California San Diego, La Jolla, California 92093, United States; ⊥ Materials Science and Engineering Program, University of California San Diego, La Jolla, California 92093, United States; # Department of Electrical and Computer Engineering, University of California San Diego, La Jolla, California 92093, United States

## Abstract

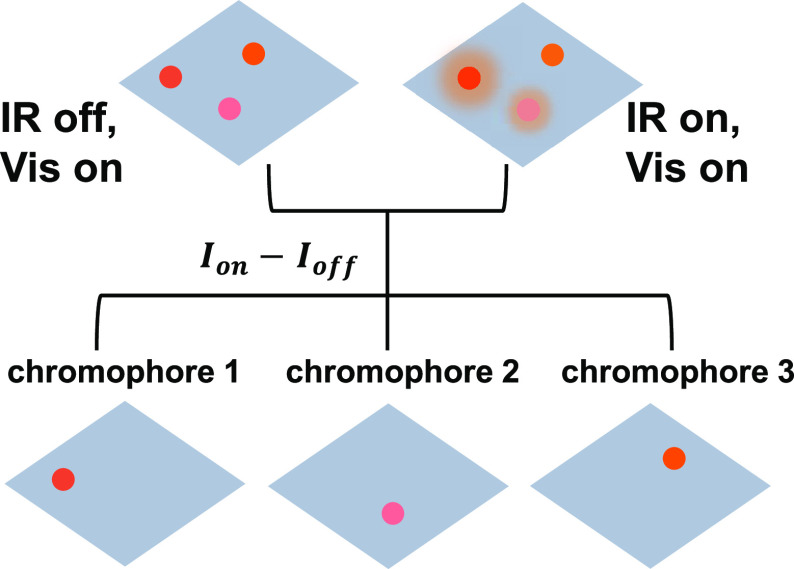

Photoluminescence (PL) imaging has broad applications in visualizing biological activities, detecting chemical species, and characterizing materials. However, the chemical information encoded in the PL images is often limited by the overlapping emission spectra of chromophores. Here, we report a PL microscopy based on the nonlinear interactions between mid-infrared and visible excitations on matters, which we termed MultiDimensional Widefield Infrared-encoded Spontaneous Emission (MD-WISE) microscopy. MD-WISE microscopy can distinguish chromophores that possess nearly identical emission spectra via conditions in a multidimensional space formed by three independent variables: the temporal delay between the infrared and the visible pulses (*t*), the wavelength of visible pulses (λ_vis_), and the frequencies of the infrared pulses (ω_IR_). This method is enabled by two mechanisms: (1) modulating the optical absorption cross sections of molecular dyes by exciting specific vibrational functional groups and (2) reducing the PL quantum yield of semiconductor nanocrystals, which was achieved through strong field ionization of excitons. Importantly, MD-WISE microscopy operates under widefield imaging conditions with a field of view of tens of microns, other than the confocal configuration adopted by most nonlinear optical microscopies, which require focusing the optical beams tightly. By demonstrating the capacity of registering multidimensional information into PL images, MD-WISE microscopy has the potential of expanding the number of species and processes that can be simultaneously tracked in high-speed widefield imaging applications.

## Introduction

Photoluminescence (PL) imaging techniques based on spontaneous emission of photons have achieved single-molecule level sensitivity^1,2^ and subdiffraction spatial resolution,^3^ becoming vital tools for fields from bioimaging^4,5^ to material characterization.^6^ However, an intrinsic limitation is that the broad PL emission spectra of chromophores can easily congest the one-dimensional visible wavelength range.^7^ As a consequence, PL images are limited to only a few independent color channels for tracking different chemical or biological entities simultaneously. Many samples are complex systems. For instance, in a cellular environment, there could be numerous different proteins and RNAs with distinct functions coexisting and interacting with each other. It is necessary to spatially map all of their locations and follow their interactions. One existing approach is to take many imaging rounds where a few types of species are labeled in each round.^8,9^ To improve the throughput and speed in multiplexed PL imaging, it is possible to simultaneously image multiple chromophores with overlapping PL spectra and differentiate them from each other through other identities (such as encoding chemical information), thereby enabling the observation of complex interactions among labeled species.

To encode images with rich chemical information, imaging methods involving other molecular degrees of freedom have been developed. Vibrational chemical imaging methods based on Raman microscopy^10−12^ and infrared (IR) photothermal microscopy^13^ are highly successful in label-free applications. The Raman scattering or IR absorption of molecular vibrational modes reports molecular structures and local chemical environments. The spectral line width of vibrational modes is generally much narrower than that of PL emission, allowing a large number of chromophores with well-separated spectral lines to be simultaneously used in labeling and multiplexed imaging applications. These vibrational imaging techniques naturally attain the ability to resolve chemicals through vibrational fingerprints.

Recently, techniques are emerging to encode vibrational information into fluorescence, a specific form of PL emission.^14−20^ Methods encoding chemical information into fluorescence images could complement well-established label-free chemical imaging methods such as photothermal and stimulated Raman scattering microscopy. These techniques combine both the sensitivity of fluorescence detection and the chemical information on molecular vibrations. For instance, fluorescence-detected mid-infrared photothermal microscopy^19,20^ is based on modulating fluorescence signals by the change of the temperature around fluorophores following vibrational relaxation. While photothermal methods are very sensitive and useful, they are intrinsically based on the optical response of a fluorophore to changes in the matrix environment, making their sensitivity dependent on the properties of the matrix.^21,22^ Nonlinear optical methods resonantly exciting multiple transitions of a molecule have enabled encoding processes using the inherent nonlinear optical response of dye molecules.^14^ Using the stimulated Raman transition to excite vibrational modes and encode a fluorophore’s emission intensity, stimulated Raman excited fluorescence microscopy^17,23^ has been demonstrated as a powerful multiplexed PL imaging method with single-molecule level sensitivity under ambient conditions. However, nonlinear optical microscopies typically operate by focusing optical beams tightly onto the sample of interest, since the cross sections of higher-order nonlinear interactions are orders of magnitude lower than first-order absorption cross sections. This imaging condition has limited most nonlinear optical microscopies to the confocal geometry, rather than the widefield geometry with a field of view larger than tens of microns.

Although difficult, it is important to encode widefield PL images with additional chemical information via nonlinear optical processes. Widefield microscopies have certain intrinsic advantages such as lower photodamage and faster imaging speed over a large field of view than confocal microscopies,^24,25^ enabling applications such as tracking cellular dynamics^25^ and fast energy dissipation processes in semiconductors.^26^ For multiplexed PL images encoded with a large amount of information, data are often collected over a set of different imaging conditions such as excitation frequencies.^12,17^ As such, a multiplexed data collection process can be time-consuming, the widefield mode could be useful for fast multiplexed PL microscopies.

A general encoding strategy is to excite other degrees of freedom in addition to the linear electronic transitions to modulate the electronic absorption and/or the subsequent PL emission process, as demonstrated in prior studies of nonlinear spectroscopy.^27−30^ The nature of the widefield imaging mode demands these additional excitation processes to possess large cross sections so that they are compatible with the condition of lower photon flux. In this work, we showed that with ultrashort femtosecond mid-IR pulses, two types of IR-visible nonlinear interactions are feasible for IR-encoded widefield PL imaging. The first type is exciting molecular vibrations through the linear absorption of a mid-IR photon that further modulates the electronic absorptions. Linear mid-IR absorption typically has a much larger cross section than nonlinear vibrational excitation such as Raman-based processes.^13,14^ The IR-visible double-resonance process,^14^ during which a dye molecule sequentially absorbs a mid-IR photon and then a visible photon, can encode PL signals in the widefield configuration.^16,31−33^ The second type of IR-visible nonlinear interaction is based on the strong electric field of femtosecond mid-IR pulses, which can reach the order of megavolts per centimeter (MV/cm) due to the high peak power and the relatively long wavelength.^34,35^ Such electric fields can ionize excitons in semiconductor materials, affecting the PL intensity of quantum dot (QD) emitters.^36^

**Figure 1:**
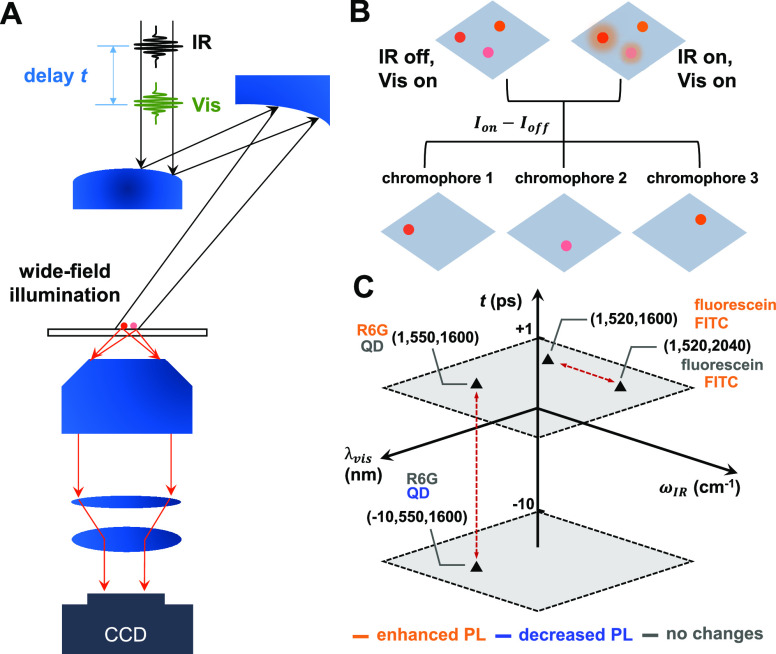
Principles of MD-WISE microscopy. (A) Schematic illustration for MD-WISE microscopy, positive delay of *t* denotes that the IR pulse arrives earlier than the visible pulse. (B) Intensity of PL signals generated following the visible excitation pulse can be encoded by the optical frequency of the IR pulse or the delay *t*. By taking the difference of PL intensities between images acquired with (*I*_on_) or without the IR pulse (*I*_off_), various chromophores can be distinguished apart even if their emission spectra are nearly identical. (C) Change of a chromophore’s PL induced by the IR pulse, either there is no change (gray), or increased PL (orange) or decreased PL (blue), is a function of independently tunable variables expressed as the three orthogonal axes: visible excitation wavelength λ_vis_ (nm), IR frequency ω_IR_ (cm^–1^), and ultrafast delay *t* (ps). By choosing a condition (▲) in the three-dimensional space, pairs of chromophores with nearly identical PL spectra can be distinguished from each other, such as the pairs of QD versus R6G and FITC versus fluorescein. The two gray shaded planes are condition planes having the same delay, +1 or −10 ps, and the coordinates in brackets are expressed as (*t*, λ_vis_, ω_IR_).

Herein, we synthesize both types of processes described above to demonstrate that the action of a mid-IR pulse can encode multidimensional information in widefield PL images and thus distinguish PL chromophores with nearly identical emission spectra. We referred to this approach as the MultiDimensional Widefield Infrared-encoded Spontaneous Emission (MD-WISE) microscopy. The PL intensity of microstructures stained by different chromophores can be modulated by either the IR optical frequency ω_IR_ or the temporal delay *t* between IR and visible pulses. Conventional PL microscopy resolves chromophores within the crowded one-dimensional space of PL emission wavelength λ_vis_, whereas the MD-WISE method can resolve chromophores in a three-dimensional space defined by *t*, λ_vis_, and ω_IR_ (Figure 1). The multidimensional “color” could potentially be useful for supermultiplexed widefield PL imaging in future applications. For instance, it opens a way to develop hundreds of chromophores distinguishable in the multidimensional space and apply them simultaneously to image many coexisting chemical or biochemical species in complex systems. The potential to increase multiplexity in PL detection can accelerate the throughput of bioanalytical screening processes using PL emission signals, such as fluorescence immunoassays. We also demonstrated that an amplified kHz laser system, such as the 20 kHz system used here, with sufficient pulse energy can enable nonlinear IR-visible interactions under the widefield imaging condition, adding to the toolbox of nonlinear optical imaging conventionally based on laser systems with MHz repetition rates.

## Experimental Section

### Linear Optical Measurements

Diffuse reflectance infrared Fourier transform spectroscopy (DRIFTS) measurements were acquired on a Thermo Fisher Nicolet iS10 spectrometer by mixing the samples with a KBr matrix. Linear UV/visible absorption spectra of stained beads were acquired on a Cary 60 spectrometer. Linear PL emission spectra of stained beads were acquired on a Hamamatsu Quantaurus-QY C11347 spectrometer. For UV/visible and PL measurements of the stained microbeads, the microbeads were sandwiched between two No.5 thin coverslips (Zeiss, 170 ± 5 μm) with transparent fluorolube oil to minimize scattering. Bright-field white light images and red-channel PL images of stained cells on No.5 coverslips were acquired using a BZ-X710 microscope (TRITC red channel: excitation filter 540/25 nm, emission filter 605/70 nm, center/width).

### Laser Systems

The femtosecond pulses used in the experiments here were produced by using a Yb-based amplifier (Carbide, Light Conversion) pumping an optical parametric amplifier (Orpheus-One, Light Conversion) system. The repetition rate of the laser was set to be 20 kHz. The mid-IR pulse was produced by the difference frequency generation crystal in the optical parametric amplifier, and the frequency center was tuned within the range of 1600–2100 cm^–1^ depending on the need. The spectral full width at half-maximum (fwhm) of the mid-IR output was ∼60 cm^–1^. The reported center frequency and bandwidth of the tunable mid-IR pulses were calibrated following an upconversion process. The Yb-based amplifier produced a fundamental pulse centered at 1025 nm, which was sent through a Fabry–Perot etalon cavity (LightMachinery Inc.) to generate a narrowband upconversion spectrum centered at 1022.7 nm with an fwhm of 3 cm^–1^. The mid-IR pulse and the narrowband upconversion pulse were overlapped spatially and temporally onto a 5% Mg-doped lithium niobate crystal (MTI Corp.) to generate a visible optical frequency at the sum of the two pulses. The sum-frequency signal was characterized by a spectrograph (300 l/mm grating, Shamrock 500i, Oxford Instruments). The mid-IR frequency is determined by the frequency difference between the sum-frequency signal and upconversion beam, and the IR spectral width σ_IR_ is determined by deconvoluting the upconversion spectral width σ_UP_ from the sum-frequency spectral width σ_SFG_ using .

For the nonbiological samples, the mid-IR pulse energy was set to 0.4 μJ using a pair of a half-wave plate and a polarizer. For the imaging of cells with a larger field of view (∼30 μm), the mid-IR pulse energy used was 1.5 μJ. The visible pulse for electronic excitation was generated by spectrally filtering a white light pulse, which was generated by focusing the optical parametric amplifier’s 725 nm visible output into an yttrium aluminum garnet (YAG) crystal. For instance, the 550 ± 5 nm visible pulse was obtained by passing the white light pulse through a 550 ± 5 nm bandpass filter. The visible pulse energy after the filter was estimated to be about only 0.25 nJ, equivalent to 5 microwatts at a 20 kHz repetition rate. Thus, a long exposure time of several seconds was used for the MD-WISE imaging experiments here, even for obtaining the IR-off plain PL images, but the exposure time shall not be an intrinsic limiting factor for the MD-WISE method. The duration of the IR and visible pulses were determined as <300 fs by cross-correlation measurements. For the experiments explained below, the IR and visible pulses were combined collinearly using a customized dichroic mirror that reflects the visible pulse and transmits the IR pulse. The delay between the two pulses was controlled by a mechanized delay stage (Newport, XMS160).

### IR-Pump White Light-Probe Ultrafast Transient Absorption Experiments

R6G was dissolved in dimethyl sulfoxide-d_6_ and the solution was sandwiched between two CaF_2_ windows with a 56 μm spacer. The white light and the mid-IR pulses were collinearly focused into the solution by a Schwarzchild reflective objective (Thorlabs, LMM40X-P01, numerical aperture 0.5). The beam size of both pulses at the focus was measured as 12 ± 3 μm using a knife-edge method. This beam size shall not be confused with the adjustable field of view of MD-WISE imaging in the next section. The IR pulse was chopped by an optical chopper at 1 kHz. The white light pulse passing through the sample was collimated and attenuated before entering a spectrograph (300 lines/mm grating, Shamrock 500i, Oxford Instruments) equipped with a conventional CCD detector (Newton 920, Oxford Instruments). The CCD’s frame rate was synchronized with the chopper at 1 kHz to collect the white light spectra when the IR pulse was blocked or unblocked, and the change of optical density induced by the IR pulse, ΔA, can be calculated at each wavelength. Transient absorption spectra were acquired with a series of temporal delays.

### MD-WISE Imaging Experiments

The stained samples were placed on a No.5 thin coverslip (Zeiss, 170 ± 5 μm). The coverslip was mounted on a 2D piezo stage (MadCity Laboratories). The filtered visible pulse and the mid-IR pulse were focused onto a coverslip (thickness 170 ± 5 μm) with beads or cells by a Schwarzchild reflective objective (PIKE Technologies Inc., PN 891-0001, numerical aperture 0.7). The field of view was adjustable by varying the beam sizes of the collinear mid-IR and visible pulses. Adjusting the field of view, therefore the IR photon density, provides flexibility to control the PL modulation level and also to customize the imaging area based on the region of interest. For example, the field of view was set to 10–20 μm to achieve high modulation levels when imaging small objects such as the silica microbeads; in contrast, to image large fixed cells in one frame, we set the field of view to 30–40 μm. The PL signals emitted by the chromophores passed through the coverslip and were collected by an infinity-corrected 20× refractive objective (Zeiss, Fluar, numerical aperture 0.75). The PL signals then passed through a bandpass filter to remove the visible excitation pulse and were projected directly on the Newton 920 CCD to form widefield images with a spatial resolution of 1.6 μm (Supporting Information), which can be further improved by using an objective with a larger numerical aperture. The difference images in MD-WISE experiments were formed by subtracting the PL image collected when the IR beam was blocked by a mechanical shutter (IR off) from the PL image collected when the IR beam was unblocked (IR on). The CCD acquisition time was set to 0.5–10 s for each image collected with the IR beam on or off, depending on the brightness of the sample.

## Results and Discussion

### Principles of MD-WISE Microscopy

The general idea, basic design, and unique capability of MD-WISE microscopy are shown in Figure 1. A pair of femtosecond IR and visible pulses delayed by a controlled interval, *t*, travel collinearly and are spatially focused onto the sample of interest by a reflective objective. The spontaneously emitted PL signals, following the ultrafast interactions of the two pulses with the chromophores, are collected by a refractive objective to form a widefield image on a conventional charge-coupled device (CCD) camera (Figure 1A). The intensity of the PL image is a function of the optical frequencies of the IR and visible pulses as well as the temporal delay *t*. Thus, as shown in Figure 1B, by taking the intensity difference of the PL images acquired with or without the IR pulse, we obtain difference images revealing species of which the PL is encoded by the IR pulse through either vibrational excitation or strong field interactions, as described above.

Because the mechanisms that encode PL are time-dependent, MD-WISE realizes a three-dimensional condition space formed by the three orthogonal variables, as illustrated in Figure 1C: the IR frequency (ω_IR_), the visible wavelength (λ_vis_), and the ultrafast delay (*t*). By choosing the appropriate condition in the space, many chromophores having nearly identical PL spectra or emitting in the same PL collection wavelength range thus can be distinguished in MD-WISE. By demonstrating the capacity of registering multidimensional information into widefield PL images, MD-WISE microscopy has the potential of further expanding the number of species and processes that can be simultaneously tracked in high-speed chemical and biological imaging applications.

### IR-Encoding Mechanism 1: Infrared-Visible Double-Resonance Process

We first demonstrate one of the two mechanisms of MD-WISE microscopy: using vibrational excitation to alter the electronic absorption cross section and the subsequent PL emission of molecular dyes. This type of double-resonance process^14^ has been previously investigated in various types of experiments that utilize mid-IR pulse to encode vibrational information into fluorescence signals.^15,16,18,31,37^ An eminent example is the broadband fluorescence-encoded IR spectroscopy that operates under the confocal configuration to read out the vibrational spectrum of single coumarin molecules in the solution phase.^18,37−39^ For widefield PL imaging, early work, transient fluorescence detected IR microscopy, showed that one can use fluorescence signals to image the spatial distribution of generic C–H and N–H stretch modes of dyes at the IR frequency of ∼3000 cm^–1^.^16^ However, the broad potential of multiplexed widefield PL imaging has not been explored in this work. Below, we report the effect of vibrationally exciting functional groups linked to various sites of dye molecules and discuss how these diverse groups can be used to differentiate nearly identical PL chromophores in widefield imaging.

**Figure 2:**
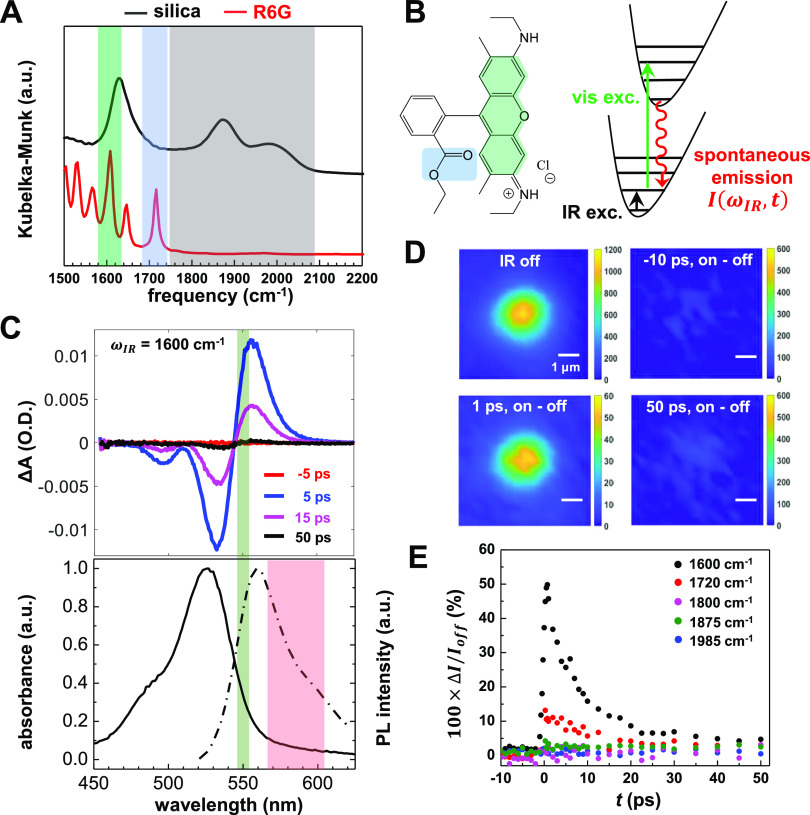
IR-encoding mechanism 1: infrared-visible double-resonance process. (A) DRIFTS results of silica microspheres and pure R6G dye. The colored shade areas mark the IR excitation frequencies covered in MD-WISE imaging experiments of silica spheres stained with R6G: the aromatic three-ring xanthene system (green), the ester group (blue), and silica absorption (gray). (B) Molecular structure of R6G, and scheme of double-resonance excitation process and the subsequent spontaneous emission of fluorescence. (C) Ultrafast transient absorption (upper panel) spectra of R6G in IR-pump-Vis-probe experiments. The steady-state absorption (solid line) and emission spectra (dashed line) of R6G-stained silica beads are in the bottom panel. The dark green area marks the excitation wavelength of the visible pulse, tuned to the excited-state absorption region in the pump–probe spectra. The red shaded area marks the collection window of the emission signal. (D) Widefield images of a silica bead, including the image collected without IR excitation and several difference images collected with the 1600 cm^–1^ IR pulse delayed at different times. The color bars represent the counts on the CCD pixels. Scale bars are 1 μm. (E) Kinetic traces of the relative difference of emission intensity in the widefield images acquired with and without IR pulse, measured with five different center frequencies of the IR pulse.

Rhodamine 6G (R6G) molecules, a prototypical fluorophore with a high fluorescence quantum yield (QY), are examined first. The solid-state linear IR absorption spectrum of R6G (Figure 2A), shows a sharp peak at 1600 cm^–1^, which is a ring stretch mode associated with the xanthene triring conjugation system (marked as green), and a peak at 1720 cm^–1^ assigned to the carbonyl stretch of the ester group (marked as blue).^40,41^ In the double-resonance excitation scheme here, the IR pulse first promotes a vibrational mode of R6G to the first excited state, ν = 1 (Figure 2B). Many vibrational modes of R6G could be involved in the vibronic couplings. Due to the shift in energy levels and changes in Franck–Condon factors,^38^ the electronic absorption spectrum of ν = 1 state is altered from that of ν = 0 ground state. Therefore, the absorption cross section of the visible photon at a specific wavelength also changes. Next, before vibrational relaxation, the visible pulse excites the molecules to the S_1_ electronically excited state. The absorption cross-section difference is read out through fluorescence as the molecule returns to the ground state S_0_. The QY of the fluorescence emission process is not affected by the IR excitation because the molecule, regardless of which vibronic state it is in immediately after the visible excitation, first relaxes to the lowest vibronic state in S_1_ before emitting the fluorescence.^42^ The vibrational relaxation occurs on the time scale of a few picoseconds while the emission of fluorescence happens in nanoseconds. Thus, the fluorescence emission does not experience vibrational excitations due to the mismatch of time scales, which is a fundamental difference from photothermal-based PL imaging methods. The IR modulation of fluorescence intensity is solely through the modulation of the electronic absorption.

We verified the effect of mid-IR excitation on the electronic absorption spectrum with IR-pump-visible-probe transient absorption experiments. The upper panel of Figure 2C displays the transient absorption spectra of R6G in dimethyl sulfoxide*-*d_6_ solution measured at several delays. The IR excitation is tuned to the xanthene stretch mode at 1600 cm^–1^. Due to the relatively large fwhm (60 cm^–1^) of the IR bandwidth, we note that the tails of adjacent modes such as the other ring stretch mode at 1650 cm^–1^ could also be excited but should have negligible effect due to the small absorptions. For short delays such as 5 or 15 ps, it is evident that there is a change in the absorbance across the visible spectrum, in contrast to the signal at negative delays or long delays such as 50 ps. Thus, the effect of IR excitation is an ultrafast double-resonance effect rather than a photothermal effect. The lack of photothermal effect could come from a combination of the relatively low laser repetition rate, compared to the commonly used MHz laser systems, as well as the relatively low IR pulse energy and large beam size deployed in the widefield imaging mode. The elimination of accumulated heating prevents complications in the processing and interpretation of images. At longer visible wavelengths, the sample exhibits a positive Δ*A* value, indicating that the visible absorption is enhanced by IR excitation; on the contrary, the visible absorbance at shorter wavelengths is reduced due to the bleaching of *v* = 0 populations. Thus, when the IR excitation is on, it leads to more absorption near 550 nm, which should lead to more fluorescence emission.

From the combined knowledge of transient and linear spectra (Figure 2C), we can select a narrow-band visible excitation wavelength centered at 550 nm (bandwidth 10 nm, green area in Figure 2C) for MD-WISE imaging experiments. At 550 nm, there is a high Δ*A* to form a bright difference PL image, with sufficient low linear absorption (thus, low background PL) to achieve a good signal-to-noise ratio. Furthermore, a 550 nm excitation has enough spectral shift from the PL detection window 585 nm (bandwidth 36 nm, red area in Figure 2C) to eliminate leakage of excitation photons. The actual excitation and PL detection wavelengths of other dye molecules such as fluorescein could vary but are selected by using the same criteria.

The next step is to examine the effect of IR excitations on widefield PL images. A set of widefield PL images of an R6G-stained microbead (diameter = 3 μm) is displayed in Figure 2D. The set includes an image collected without IR excitation and several difference images collected with the delay *t* set to −10, 1, and 50 ps, respectively, with the IR center frequency set to 1600 cm^–1^. The difference images are generated by subtracting the PL image without the IR excitation from the image with it. At the short positive delay of 1 ps, the IR pulse enhances the emission signal strongly because most of the vibrationally excited molecules have not relaxed yet. Furthermore, the difference image at 1 ps agrees with the IR-off PL image, reflecting that it faithfully reproduces the shape of the microbead and the spatial distribution of R6G. In contrast, the microbead disappears in the difference images at −10 and 50 ps since the molecules have returned to the vibrational ground state.

To characterize the vibrational dynamics and the IR frequency dependence of PL encoding, we measured the ultrafast kinetics of the PL intensity change for a series of IR excitation frequencies (Figure 2E). At a specific delay *t*, the IR-induced change of PL intensity per CCD pixel, (*I*_on_ – *I*_off_)/*I*_off_, is calculated by averaging the relative intensity change among all the CCD pixels in a 2.5 μm × 2.5 μm box that centers around the microbead. Only when the IR frequency is tuned to the vibrational modes of R6G can the PL intensity be encoded by the IR pulse. When the IR frequency center is tuned to 1800, 1875, 1985, 2050, and 2100 cm^–1^ to cover the modes of silica in the range 1800–2100 cm^–1^ (the gray box in Figure 2A), the kinetic traces in Figure 2E show no modulation or encoding signal. This indicates that exciting the silica substrate does not change the PL intensity. Thus, the IR-induced PL change originates from intramolecular processes. Additional evidence is included in Supporting Information Figures S5 and S6. The extent of PL change differs among various vibrational modes of R6G. The highest occupied molecular orbital (HOMO) and lowest unoccupied molecular orbital (LUMO) of R6G are located on the xanthene ring conjugation system. Thus, it is expected that the 1600 cm^–1^ vibrational mode of the xanthene has a stronger effect on the electronic absorption spectrum than the 1720 cm^–1^ carbonyl stretch mode of the ester group. The carbonyl stretch mode is a relatively local mode involving mostly the displacements of ester group atoms, but it could couple with the displacements of the xanthene atoms via anharmonic vibrational coupling (Supporting Information Section V). The unexpected result that nonxanthene groups can affect PL intensity relaxes the conditions of where vibrational tags can be installed on chromophores and expands the possible library of chromophores for MD-WISE imaging. Another possible explanation of the distant vibronic coupling observed here is that the vibration of the ester group dissipates into other modes that have strong vibronic coupling effects via the intramolecular vibrational energy redistribution (IVR) process. However, the IVR mechanism would cause a short growth period in the kinetics as it takes time for the initial mode to relax into other modes. The absence of growth kinetics in Figure 2E suggests that the effect of exciting the ester group at 1720 cm^–1^ may come from anharmonic coupling, as discussed above, instead of the IVR process. The lifetime of the vibronic coupling effects is around 7 ps regardless of the use of 1600 or 1720 cm^–1^ IR excitation. This could be due to that the vibrational lifetimes of the ester group and the xanthene ring mode are dominated by similar energy dissipation channels.

### IR-Encoding Mechanism 2: Strong Field Ionization of Excitons in QDs

The scope of this work is further expanded beyond molecular fluorescence to PL emission of semiconductor nanocrystals referred to as QDs. QD chromophores are bright emitters of which the PL intensity can be encoded by an ultrashort IR pulse.^36^ However, the second mechanism is distinct from mechanism 1 discussed above. The IR-induced PL change of QDs originates from the strong electric field strength of an ultrashort mid-IR pulse which can drive electrons of excited-state QDs to overcome the potential barriers in core/shell QDs, leading to events such as the discharging of trion states and the dissociation of excitons.^36^ The low frequency phonon modes of inorganic nanocrystals are off-resonant from the IR frequencies (1600–2100 cm^–1^) used in this study and thus do not interact with the IR pulse.

**Figure 3:**
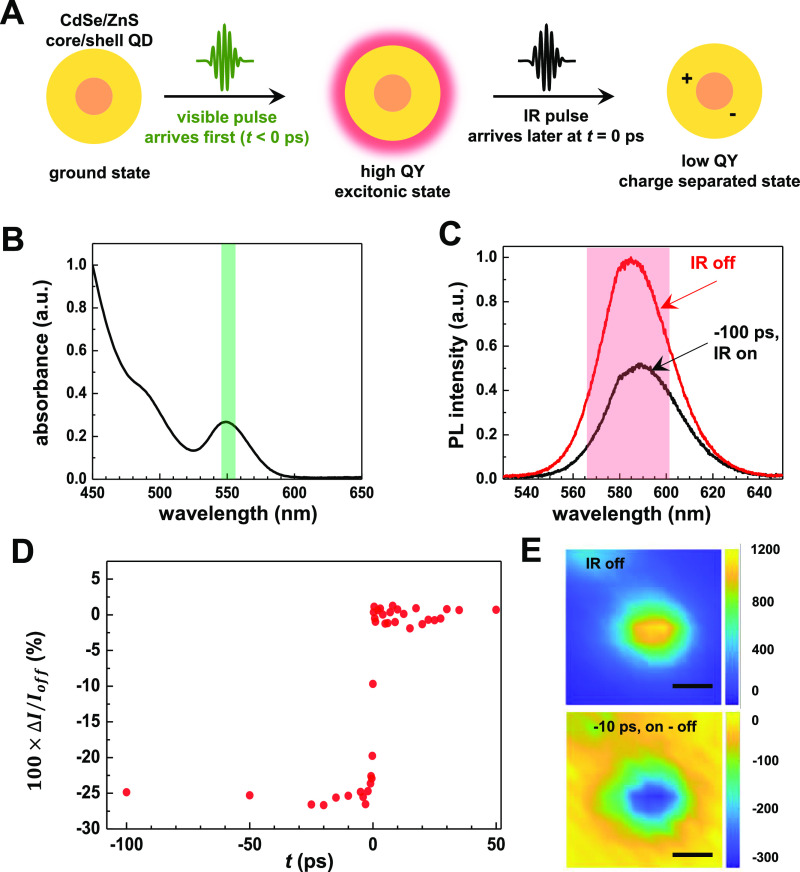
IR-encoding mechanism 2: strong field ionization of excitons in QDs. (A) Illustration of the process during which the electric field of mid-IR pulse quenches the PL emission of a QD following visible pulse excitation. (B) Visible absorption spectrum of the QD-stained silica microspheres, and the visible excitation wavelength (green) in MD-WISE imaging. (C) PL spectra of the QD-stained silica microspheres, showing that IR pulse (2100 cm^–1^) reduces the emission intensity significantly and redshifts the emission spectrum. The red area represents the collection window used for MD-WISE imaging. (D) Kinetic trace of the relative difference of emission intensity in the widefield images induced by the IR pulse (2100 cm^–1^). (E) Widefield image of a silica bead without IR excitation and a difference image with the IR pulse delayed to −10 ps show that the PL signal is significantly quenched by the IR. Scale bars are 2 μm.

The encoding mechanism employed here is illustrated in Figure 3A. First, the visible pulse excites the CdSe/ZnS core/shell QD into an excitonic state. If no IR pulse arrives, the exciton in QD would eventually emit a photon with a certain QY. If an IR pulse arrives before the emission occurs, the high electric field strength, ∼ 50 MV/cm for the conditions applied here, can dissociate the electron–hole pair of an exciton into separated charge carriers. The charge-separated state has a significantly lower QY for PL emission than the excitonic state,^43,44^ and therefore, the action of the IR pulse quenches the PL intensity of a QD. This strong field effect of the IR pulse on QDs shares some similarities with the strong field ionization in atomic and molecular optical physics where an intense long-wavelength laser pulse favors ionizations in atoms and molecules through tunneling ionizations and can drive the electrons further from the ionized atoms to mitigate recombination.^45,46^

We used a 550 ± 5 nm (green area in Figure 3B) visible pulse to excite the first-excitonic absorption feature of the CdSe/ZnS QDs, and then, a strong IR pulse arrives. The center frequency of the IR pulse is tuned to 2100 cm^–1^ (fwhm = 60 cm^–1^), but the precise value can vary since the IR frequency is off-resonant: what matters is the electric field strength.^36^ In Figure 3C, we plot the PL spectra of QD-stained silica beads acquired with and without the IR pulse. The temporal delay *t* is −100 ps. It is evident that the PL intensity drops significantly and the PL spectrum red-shifts due to the formation of low QY charge separated states following the action of the IR pulse. As shown in Figure 3D,E, the quenching of PL intensity measured from MD-WISE images of QD-stained silica beads starts sharply as delay *t* becomes negative, i.e. when the IR pulse arrives later than the visible pulse. The relative change of PL intensity does not vary much from *t* = −1 to −100 ps since the exciton lifetime of a QD is typically on the order of nanoseconds.^44^

Phenomenologically, this IR-encoding mechanism differs significantly from mechanism 1 for dye molecules, as it requires an opposite pulse sequence. The fact that the PL emission of molecular dyes is not subject to the electric field action of the IR pulse at negative delays could be due to the fact that the molecular excitons are more spatially confined and molecular energy levels are more discretely distributed than those of QDs. In MD-WISE imaging, we will take advantage of the orthogonal behaviors of QDs and molecular dyes to demonstrate the concept of distinguishing chromophores by solely varying the delay *t*. This concept is indicated by the vertical red arrow in Figure 1C.

Thus, up to now, we have presented two distinct mechanisms to encode PL with ultrashort mid-IR pulses, which enable three-dimensional multiplexing (time, IR, and visible frequencies) of PL imaging – the foundation of MD-WISE imaging. Next, we demonstrate the applications of both mechanisms.

### Distinguishing Molecular Dyes by Tuning the IR Frequency

**Figure 4:**
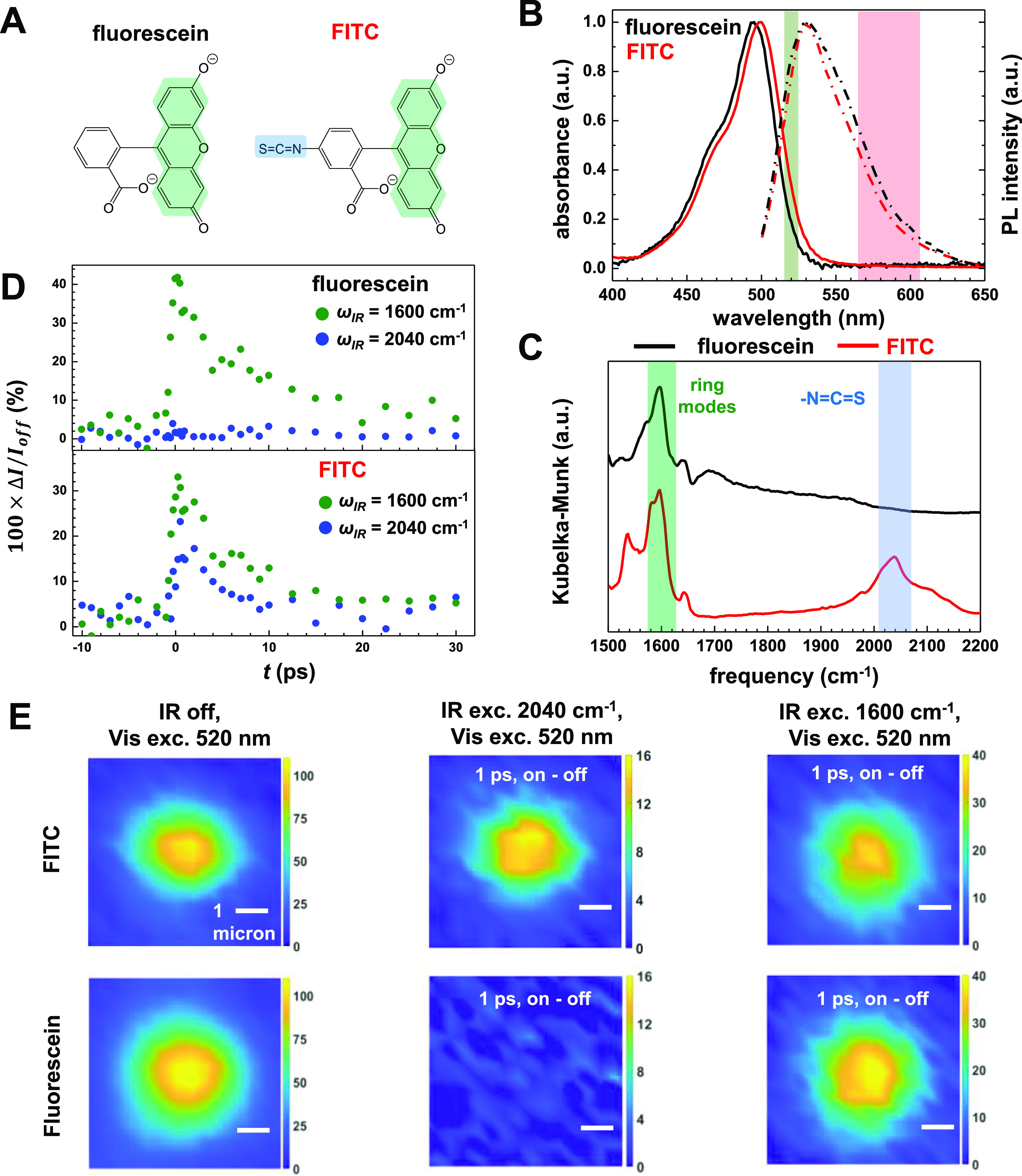
Distinguishing molecular dyes by tuning the IR frequency. (A) Molecular structures of fluorescein and FITC anions. The green shade area marks the aromatic rings responsible for fluorescence and the blue shade area marks the isothiocyanate group. (B) Steady-state absorption (solid lines) and emission (dashed lines) spectra of FITC and fluorescein adsorbed on silica beads. The green area marks (520 ± 5 nm) the excitation wavelength of the visible pulse, and the red area (585 ± 18 nm) marks the collection window of fluorescence signals in MD-WISE imaging. (C) DRIFTS spectra of FITC and fluorescein. Tuning the IR center frequency to 1600 cm^–1^ (green) or 2040 cm^–1^ (blue) excites the common vibrational modes of the xanthene ring or the isothiocyanate group unique to FITC, respectively. (D) Ultrafast kinetic traces of the relative difference of emission intensity in the widefield images induced by the IR pulse, measured using silica beads stained with FITC and fluorescein at 1600 and 2040 cm^–1^. (E) Responses of stained beads under different conditions. Left column is the fluorescence image of a single 3 μm bead without an IR pulse. The middle and right panels are the difference images at *t* = +1 ps acquired at 1600 and 2040 cm^–1^, respectively. All scale bars are 1 μm.

We first demonstrate the distinguishing of two chromophores with nearly identical absorption and PL spectra using mid-IR vibrational excitations. The molecular dianion structures of fluorescein-5-isothiocyanate (FITC) and fluorescein are displayed in Figure 4A, and they only differ by one functional group: the isothiocyanate (−N=C=S) group. As shown in Figure 4B, the two molecules have nearly identical electronic absorption and emission spectra, making it difficult to distinguish them by choosing specific excitation or emission wavelengths. In contrast, MD-WISE microscopy should distinguish FITC from fluorescein by exciting the asymmetric stretch mode of the −N=C=S group. The DRIFTS results of FITC and fluorescein (Figure 4C) show that they both have the generic xanthene ring stretch at 1600 cm^–1^ (the green shade area),^47^ and FITC has a unique isothiocyanate stretch mode at 2040 cm^–1^ (the blue shade area).^48^ The 2040 cm^–1^ mode is accompanied by side bands that could be assigned to Fermi resonances between the −N=C=S stretch mode and the low-frequency combination modes of the rings in FITC at ∼1000 cm^–1^.^48^ Although the −N=C=S group is not directly attached to the xanthene rings, its stretch mode may affect PL intensity of the FITC dye through coupling to the displacements of atoms in xanthene rings via Fermi resonances.^49,50^

The effects of different vibrational modes on PL images are then investigated. The ultrafast kinetics of the PL intensity change of stained silica beads are plotted in Figure 4D. When the IR frequency center is tuned to 1600 cm^–1^ with an fwhm of 60 cm^–1^, both FITC and fluorescein show significant IR-induced PL intensity change over the delay time range of 0–10 ps. When the IR frequency range is tuned to 2040 cm^–1^ with an fwhm of 60 cm^–1^, only FITC shows a PL intensity change. Comparing the percentage of PL change at fixed delays in the lower panel of Figure 4D, the xanthene ring modes at 1600 cm^–1^ have larger effects on the PL intensity than the −N=C=S stretch mode. This is expected because xanthene rings are directly responsible for the visible absorption and emission properties. However, the −N=C=S stretch mode encoding can be used to distinguish these two molecules.

We now demonstrate differentiating FITC and fluorescence in microbeads by IR excitations in widefield MD-WISE images in Figure 4E. The left column shows the PL images without applying the IR pulse. The middle and the right columns show the difference images acquired at a short delay time of 1 ps using IR center frequencies of 1600 and 2040 cm^–1^, respectively. Using 2040 cm^–1^ IR excitation, it is evident that only the FITC-stained bead appears in the difference image, while the fluorescein-stained bead is invisible. In contrast, the 1600 cm^–1^ excitation makes beads dyed by both molecules visible. Thus, in addition to the example of the ester group in the R6G molecule discussed above, the −N=C=S group on FITC marks another case where vibration of a functional group not attached to the xanthene rings can affect the PL emission. The results presented here demonstrate that dyes with nearly identical emission spectra can be distinguished through their distinction in vibrational modes. The large IR absorption cross section enables the IR-encoded PL images to be acquired in a widefield manner. As shown in the cell imaging experiments using relatively low pulse energies, 1.5 μJ of IR and 0.25 nJ of visible pulses, we can directly acquire PL images with a field of view of ∼30 μm without raster scanning or stitching images together.

### Distinguishing QDs from Molecular Dyes by Tuning the Ultrafast Time Delay between Pulses

**Figure 5:**
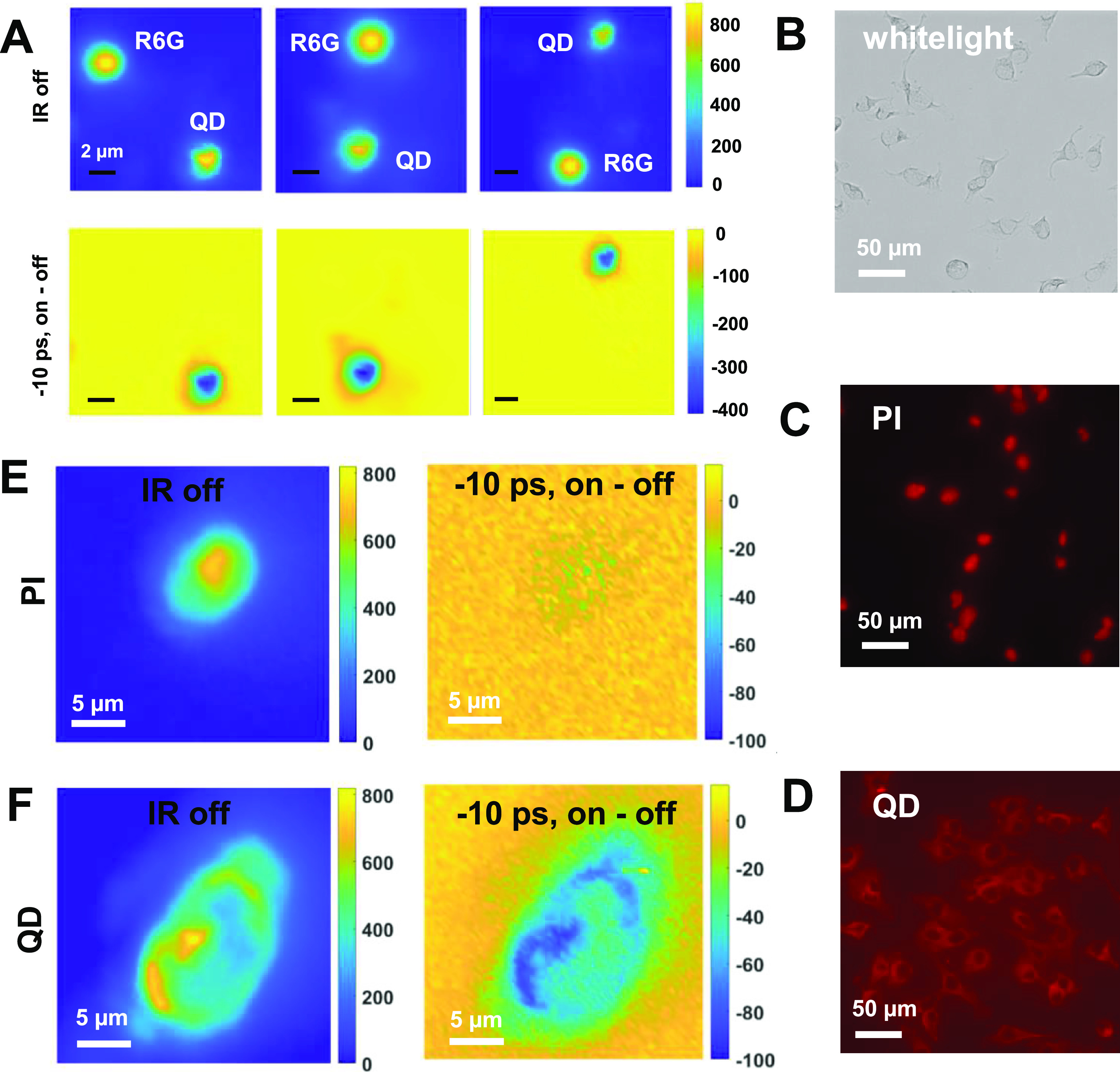
Distinguishing QDs from molecular dyes by tuning the ultrafast delay between pulses. (A) Top rows show three IR-off images of 3 μm silica beads stained with R6G comixed with 2 μm silica beads stained with QDs. In the bottom row, the difference images acquired using an IR frequency centered at 1600 cm^–1^ with a delay set to −10 ps show that the QD beads can be distinguished from the R6G beads. The color bars represent the counts on CCD pixels. Scale bars are 2 μm. (B–D) White light bright field image of fixed cells (B), and red-channel PL images of PI-stained (C) and QD-stained (D) fixed cells. (E) Widefield PL image of a PI-stained cell without using the IR pulse (left) and the difference PL image at −10 ps (right) acquired using MD-WISE microscopy. (F) Widefield PL image of a QD-stained cell without using the IR pulse (left) and the difference PL image at −10 ps (right) acquired using MD-WISE microscopy. All the images in panels E and F are acquired using IR frequency centered at 2100 cm^–1^, visible excitation at 550 ± 5 nm, and PL collection wavelength range of 585 ± 18 nm.

The PL emission of QDs and molecules requires opposite pulse sequences to encode the IR-visible interactions. In MD-WISE imaging, we take advantage of the orthogonal behaviors of QDs and molecular dyes and demonstrate the concept of distinguishing chromophores by solely varying the delay *t*. In Figure 5A, we show PL images of mixed silica beads. The smaller 2 μm beads are stained with QDs and the larger 3 μm beads are stained with R6G molecules. We can capture both types of beads within the same view without rastering. The PL collection wavelength window covers the emission spectra of both QDs and R6G molecules. The IR frequency center is tuned to the 1600 cm^–1^ xanthene ring mode of R6G, while there is no frequency requirement to encode the PL of QDs. At *t* = −10 ps, the R6G-stained beads disappear from the difference images in the bottom row of Figure 5A, while QD-stained beads remain due to the significant quenching of the PL intensity by the IR pulse. The orthogonal encoding behaviors of QDs and molecular dyes suggest that, for almost any emission color, we may choose a QD with a matching bandgap to act as the counterstain for the molecular dyes that emit the same color in PL imaging.

QD chromophores are bright PL emitters for biological imaging.^51−53^ We last demonstrated the proof-of-principle application of MD-WISE in differentiating QDs and molecule dyes in biological samples. The white light bright field images and conventional PL images of fixed human breast cancer cells are shown in Figure 5B–D. The cells are either stained by the QDs coated with streptavidin to visualize the cell membrane or the molecular dye propidium iodide (PI) to visualize the cell nucleus. The QDs used for staining cell membranes have nanostructures similar to the ones for staining silica beads in Figures 3E and 5A, supported by their similar emission peaks at ∼585 nm. The difference is the surface ligands, which lead to different binding affinity to these QDs. The cell-staining QDs are coated with streptavidin, allowing binding to the biotin of cell membranes, while silica-staining QDs are coated by organic amine molecules to coat silica surfaces.

Although the QDs and PI dyes are separated in the cells by their binding specificities to different biological structures, both chromophores emit in the red channel of a conventional widefield PL microscope and thus cannot be simply distinguished by PL emissions if we do not have prior knowledge of the binding properties. To differentiate them, we applied the 2100 cm^–1^ (fwhm = 60 cm^–1^) IR excitation pulse following the visible pulse and detected the change in PL emissions of both the QDs and the PI dyes. The IR frequency is chosen in the cell-silent IR region to avoid excess IR absorption by water and biomolecules in the cells. In Figure 5E, although the regular PL image shows the cell nucleus, the difference image acquired at −10 ps only shows a blank, indicating that there is no IR-induced PL change at this negative delay, verifying that the cell nucleus is stained by PI dyes. In Figure 5F, the −10 ps difference image reproduces well the shape of the cell membrane, as seen in the PL image acquired without the IR pulse, verifying that QDs coated with streptavidin only stain the cell membrane. The results demonstrate that MD-WISE microscopy can resolve QD and molecular dyes as counterstains from each other by purely optical means even though these chromophores emit at the same detection channel.

**Figure 6:**
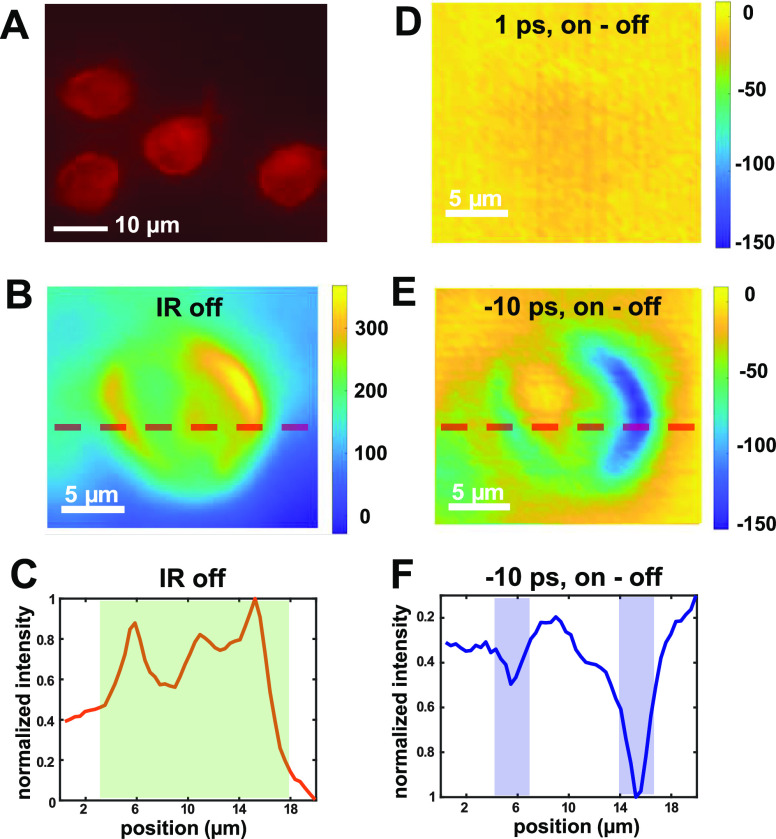
Distinguishing QD-stained cellular membranes from PI-stained nucleic acids in a single cell by tuning the ultrafast delay between IR and visible pulses. The color bars represent the counts on CCD pixels. (A) Red-channel PL images of cells costained by both PI dyes and QDs. (B) MD-WISE image of a costained cell when IR pulse is blocked. The dashed horizontal line indicates the location where linecut intensity analysis was performed. (C) Normalized intensity plot aligned along the line in (B), showing signals throughout the cell (shaded area). (D) MD-WISE difference PL image with delay *t* set to 1 ps, showing no contrast for PI and QD stains. (E) MD-WISE difference PL image with delay *t* set to −10 ps, showing negative contrast for QD-stained membranes. The dashed horizontal line indicates the location where linecut intensity analysis was performed. (F) Normalized intensity plot aligned along the line in (E), showing signal peaks at the location of membranes (shaded area). All the MD-WISE images in panels B, D, and E were acquired using IR frequency centered at 2100 cm^–1^, visible excitation at 550 ± 5 nm, and PL collection wavelength range of 585 ± 18 nm.

We also examined the cells costained by both the QD and PI chromophores to demonstrate the ability of MD-WISE microscopy of distinguishing cellular components in the same cell. As shown in Figure 6A, both the interior of the cells and cellular membranes appear as stained under the red channel of a conventional widefield PL microscope. Figure 6B shows the MD-WISE image of a costained cell when the IR pulse is blocked, which appears similar to the cells shown in Figure 6A. To clearly visualize various components in the image, we perform a linecut through the image and analyze the normalized pixel intensity across the image. The normalized intensity plot in Figure 6C shows that PL signals are observed throughout the cell, i.e., including the cell nucleus and membranes. In contrast, when we apply the 2100 cm^–1^ IR pulse, the MD-WISE difference PL image at delay *t* = −10 ps selectively resolves the QD-stained cell membranes. The membranes show negative contrast in Figure 6E due to the PL quenching effect of the IR pulse, while the nucleic acids stained by PI show no contrast. The normalized linecut intensity plot in Figure 6F shows only two major peaks corresponding to the boundaries of the QD-stained membranes. As a control experiment, the MD-WISE difference PL image at *t* = 1 ps in Figure 6D shows only the expected blank image, since the IR pulse neither is tuned to the resonant vibrational frequency of the PI molecular dye nor is able to affect the emission intensity of QDs when it arrives earlier than the visible pulse. Thus, the MD-WISE images can be used to differentiate various components of a single cell stained by chromophores emitting in the same wavelength range and are nearly free from any photothermal background. In future applications, one color channel can be used to simultaneously monitor the shape and status of both cell membranes and the nucleus while saving other color channels for purposes such as imaging the glycosylation on the cell surface or drug delivery across membranes. Using the multidimensional “colors” in MD-WISE imaging thus allows future discovery of intricated connections between biological events and the complex interplays among biomolecules.

## Concluding Remarks

We have demonstrated here the concept of distinguishing chromophores in widefield PL imaging using independently tunable parameters such as the IR frequency, visible wavelength and the ultrafast temporal delay in a three-dimensional condition space. The orthogonal responses of chromophores to an ultrashort IR pulse shall not be limited to the demonstrated case here and can, in principle, be applied to distinguish other chromophores that have significantly overlapping emission spectra. Functional groups with distinct vibrational frequencies, such as isothiocyanate, nitrile, or azide groups, can be installed on bright fluorophores, such as xanthene or cyanine dyes.

The effectiveness of vibronic coupling between vibrational tagging groups and electronic transitions has a direct impact on the imaging quality of the MD-WISE method. Vibrational tags covalently bonded to the conjugated emissive ring of dye molecules tend to have strong vibronic coupling effects. However, this work also shows that there is much flexibility in the chemical sites where vibrational tags can be installed. Vibronic coupling over long distances, such as the effect of the ester group on the emission of R6G and the effect of the isothiocyanate group on the emission of FITC, is feasible. The future design of dye toolkits for MD-WISE imaging depends on understanding vibronic coupling mechanisms and discovering new coupling pathways, as it can further expand the approach to distinguish chromophores. For example, using vibrational modes with different enough lifetimes can allow a significant portion of one chromophore to remain in the vibrational excited state and be seen by MD-WISE while the other chromophore has largely returned to the vibrational ground state.^54^ Different vibrational lifetimes of the same dye may also be used to infer the differences in local chemical environments within cells, tissues, or fabricated chemical devices. These extensions require key knowledge of the IVR pathways. Besides molecular probes for MD-WISE imaging, it is also feasible to design QD probes that show tailored responses to the electric field of IR pulses.

We note our choice of femtosecond IR pulses over picosecond IR pulses in MD-WISE experiments. Femtosecond pulses are critical for generating the high electric field needed for modulating the PL of QDs. Furthermore, femtosecond pulses offer a better chance to distinguish vibrational modes of which the lifetime is often only a few picoseconds. It may appear that picosecond IR pulses possess a narrower bandwidth and better spectral selectivity than femtosecond IR pulses. However, equipment such as a simple Fabry–Perot cavity or an acoustic-optical modulator^55,56^ can convert the broad spectrum of a femtosecond IR pulse to a narrowband spectrum or even an arbitrary spectrum, offering a general strategy to encode the IR excitation process. For instance, the bandwidth of a femtosecond IR pulse can be edited to only excite one vibrational mode when the full bandwidth can cover multiple modes.^57^

A possible direction opened by this work is multiplexed widefield PL imaging without using exogeneous PL chromophore labels but using intrinsic PL such as the autofluorescence of the sample. Being able to image the intrinsic chemical species in a microscopic sample is central to our capacity to understand and interact with physical and biological systems. However, for instance, the autofluorescence of multiple species in biological samples often have overlapping emission spectra.^58,59^ To enable multiplexed chemical PL imaging, the MD-WISE method can either use the intrinsic vibrational modes of fluorescent biomolecules or the vibrational modes of modified molecules such as a tryptophan^60^ tagged with a small, nonperturbative vibrational label. We note that the vibrational absorption of water causes the penetration depth of the mid-IR beam in water to be typically less than a few tens of microns. This however does not limit the biological application of MD-WISE imaging. Widefield imaging is most useful for thin samples such as cells and sectioned tissues that do not have severe water absorption background. Aqueous buffer solutions can be confined between coverslips to form thin liquid films that are penetrable by the mid-IR beam. Furthermore, in the epifluorescence imaging geometry, the short IR penetration depth in water can be utilized to confine the focusing depth of IR and thus improve the three-dimensional imaging contrast and resolution of the IR-modulated PL signal of MD-WISE imaging.

The current MD-WISE microscopy was demonstrated using a conventional CCD without electron-multiplying (EM) capacity, and the visible excitation power is only ∼0.25 nJ per pulse. Thus, there is significant space for improvements in sensitivities, which can be straightforwardly achieved by more sensitive detectors and more powerful visible excitation lasers. For PL detection in the visible region, single particle or single molecule level sensitivity can nowadays be achieved routinely using an EM-CCD or avalanche photodiode detector.^5,17,18,36^ Since the relative change of PL intensity induced by the IR pulse can reach tens of percentages at short delays without photothermal background and MD-WISE is based on the resonant excitation of the chromophore itself, the ultimate sensitivity limit of MD-WISE microscopy has the potential reach or become comparable to the single-molecule level when using highly sensitive detectors. This may in the future enable highly multiplexed super-resolution PL imaging.

Recently, an experiment similar to the encoding mechanism 1 shown here has been reported using the confocal imaging mode or with a limited field of view of ∼3 μm using picosecond 80 MHz optical parametric oscillator (OPO) systems.^61^ Since the photon flux of IR excitation is inversely proportional to the size of the illuminated area, widefield imaging requires high energy for each IR pulse that modules the PL intensity. Our MD-WISE experiments benefit from the usage of kHz optical parametric amplifier (OPA) systems. The low-repetition-rate OPA systems generally produce a much higher pulse energy than the high-repetition-rate OPO systems. Using an IR pulse energy of 1.5 μJ, we can acquire images with a field of view of ∼30 μm. Considering that many cells have dimensions similar to or smaller than 30 μm, the current widefield imaging technique has a practical importance to image a whole cell without rastering or stitching images. Higher IR pulse energy from an OPA system, e.g., 20 μJ, can potentially enable a field of view >100 μm and higher imaging speed in widefield imaging. Overall, the development of new PL probes and improvement of excitation and detection conditions for MD-WISE microscopy could enable highly sensitive multidimensional information acquisition on complex biological and chemical systems and enable the well-established kHz laser systems to contribute to biomedical imaging applications.

## Supporting Information Available

Materials and methods; additional transient absorption data and MD-WISE images; and quantum chemical analysis of vibrational modes (PDF)
