# Supplementary material for: Multidimensional Widefield Infrared-Encoded Spontaneous Emission Microscopy: Distinguishing Chromophores by Ultrashort Infrared Pulses
Source: J Am Chem Soc. 2023 Dec 12;146(3):1874–86. doi: 10.1021/jacs.3c07251 (PMC10811677; doi:10.1021/jacs.3c07251)
Supplement: Supplementary file 1 — ja3c07251_si_001.pdf [file ja3c07251_si_001.pdf]

Supporting Information for

Multi-Dimensional Widefield Infrared-encoded Spontaneous Emission Microscopy: Distinguishing Chromophores by Ultrashort Infrared Pulses

Chang Yan*[1,2,3], Chenglai Wang[1], Jackson C. Wagner[1], Jianyu Ren[1], Carlynda Lee[1], Yuhao Wan[4], Shizhen E. Wang[4], and Wei Xiong*[1,5,6]

[1]Department of Chemistry and Biochemistry, University of California San Diego, La Jolla, California 92093, United States

[2]Center for Ultrafast Science and Technology, School of Chemistry and Chemical Engineering, Shanghai Jiao Tong University, Shanghai, 200240 China

[3]Zhangjiang Institute for Advanced Study, Shanghai Jiao Tong University, Shanghai, 200240 China

[4]Department of Pathology, University of California San Diego, La Jolla, California 92093, United States

[5]Materials Science and Engineering Program, University of California San Diego, La Jolla, California 92093, United States

[6]Department of Electrical and Computer Engineering, University of California San Diego, La Jolla, California 92093, United States

*Corresponding Authors:

Chang Yan: changyan@sjtu.edu.cn

Wei Xiong: w2xiong@ucsd.edu

This PDF file includes:

Supporting text

Figures S1 to S21

Tables S1 to S7

SI References

I. Materials and staining methods.

Materials. R6G (catalog no. 83697), FITC (catalog no. F7250), fluorescein (catalog no. 46955), PI dyes (catalog no. P4170), and mesoporous silica microspheres (catalog no. 806587 for 2 microns, 806765 for 3 microns) were purchased from Sigma-Aldrich. Qdot™ 585 Streptavidin Conjugate (Invitrogen, catalog no. Q10111MP), EZ-Link™ Sulfo-NHS-SS-Biotin (catalog no. A39258) were purchased from Thermo Fisher. Ammine-coated CdSe/ZnS QD aqueous solutions were purchased from NN-Labs (catalog no. HECZWA560). All chemicals and materials were used without further purification.

Staining of silica microbeads. For the staining of silica microbeads with molecular dyes, dyes were diluted into ~1 mg/mL solutions (chloroform for R6G, ethanol for fluorescein and FITC), then the silica microbeads were soaked in the solutions and filtered. For fluorescein and FITC, 2 equivalent of NaOH (dissolved in ethanol) were added to form the bright dianions (37) before adding the silica beads. For the staining using ammine-coated CdSe/ZnS QDs, QDs were diluted into 0.1 mg/mL aqueous solutions, then the silica microbeads were soaked in the solutions, then the water was removed by a rotary evaporator.

Staining of fixed cells. i. QD staining. MDA-MB-231 human breast cancer cells (HTB-26) were obtained from American Type Culture Collection (ATCC; Manassas, VA) and cultured in Dulbecco's Modified Eagle's medium (Gibco; Waltham, MA) supplemented with 10% fetal bovine serum (Sigma-Aldrich; St. Louis, MO) at 37°C in a humidified incubator with 5% CO_2. The cells growing on coverslips at ~60% confluence were washed three times with phosphate-buffer saline (PBS) solutions at pH = 8.0. Then 250 µL PBS containing 0.5 mg/mL EZ-Link™ Sulfo-NHS-SS-Biotin was added. The cells were incubated at room temperature for 30 minutes, and then washed three times with ice-cold PBS. Cells were then fixed in 100% cold methanol for 10 min at -20°C, and then washed three times with PBS (5 min/wash). For the subsequent binding with biotin, we added 250 µL PBS containing 40 nM Qdot™ 585 Streptavidin Conjugate and incubated the cells for 1 hour at room temperature. The cells were then washed three times with PBS and then rinsed with water. **ii. PI staining.** The cells growing on coverslips at ~60% confluence were washed three times with PBS solutions at pH = 8.0. Cells were then fixed in 100% cold methanol for 10 min at -20°C, washed three times with PBS (5 min/wash), and then rinsed with 2× saline sodium citrate (SSC, containing 0.3 M NaCl, 0.03 M sodium citrate, pH 7.0) solution. Then, 250 µL of 500 µM PI dye aqueous solution was added to the cells for an incubation of 15 minutes. Cells were rinsed three times with 2X SSC and then with water. **iii. QD-PI co-staining.** MDA-MB-231 cells growing on coverslips were labeled with EZ-Link™ Sulfo-NHS-SS-Biotin and fixed in 100% cold methanol as described in the section i. QD staining above. The fixed cells were then washed three times with PBS, rinsed with 2× SSC, and incubated with 250 µL PI dye solution as described in the above section ii PI staining. The concentration of PI solution used here was 75 µM instead of 500 µM. After washing with 2× SSC and then with PBS, the cells were incubated with 250 µL PBS containing 40 nM Qdot™ 585 Streptavidin Conjugate for 1 hour at room temperature. The co-stained cells were then washed three times with PBS and then rinsed with water prior to imaging.

II. Additional data on rhodamine 6G (R6G).

(a). Solution phase measurements.

Fig. S1 shows the infrared-pump-visible-probe transient absorption (TA) results of R6G dissolved in chloroform and in dimethyl sulfoxide-d$_6$ (DMSO-d$_6$) solutions. The solutions are sandwiched between two infrared-transparent CaF_2 windows with 56 microns in thickness and optical density of ~0.2 at 540 nm. Clear features of excited-state absorption and ground-state bleaching are observed. The ultrafast kinetics of the visible-region absorbance change induced by the 1600 cm^{-1} infrared (IR) pump are sensitive to the solvent environment. In deuterated DMSO, the kinetics only exhibit a decaying pattern, whereas the decay kinetics in chloroform exhibit a more complex pattern. The difference in decay patterns might be attributed to change of Franck-Condon factors and intramolecular vibrational energy redistribution (IVR) of R6G in different solvents.

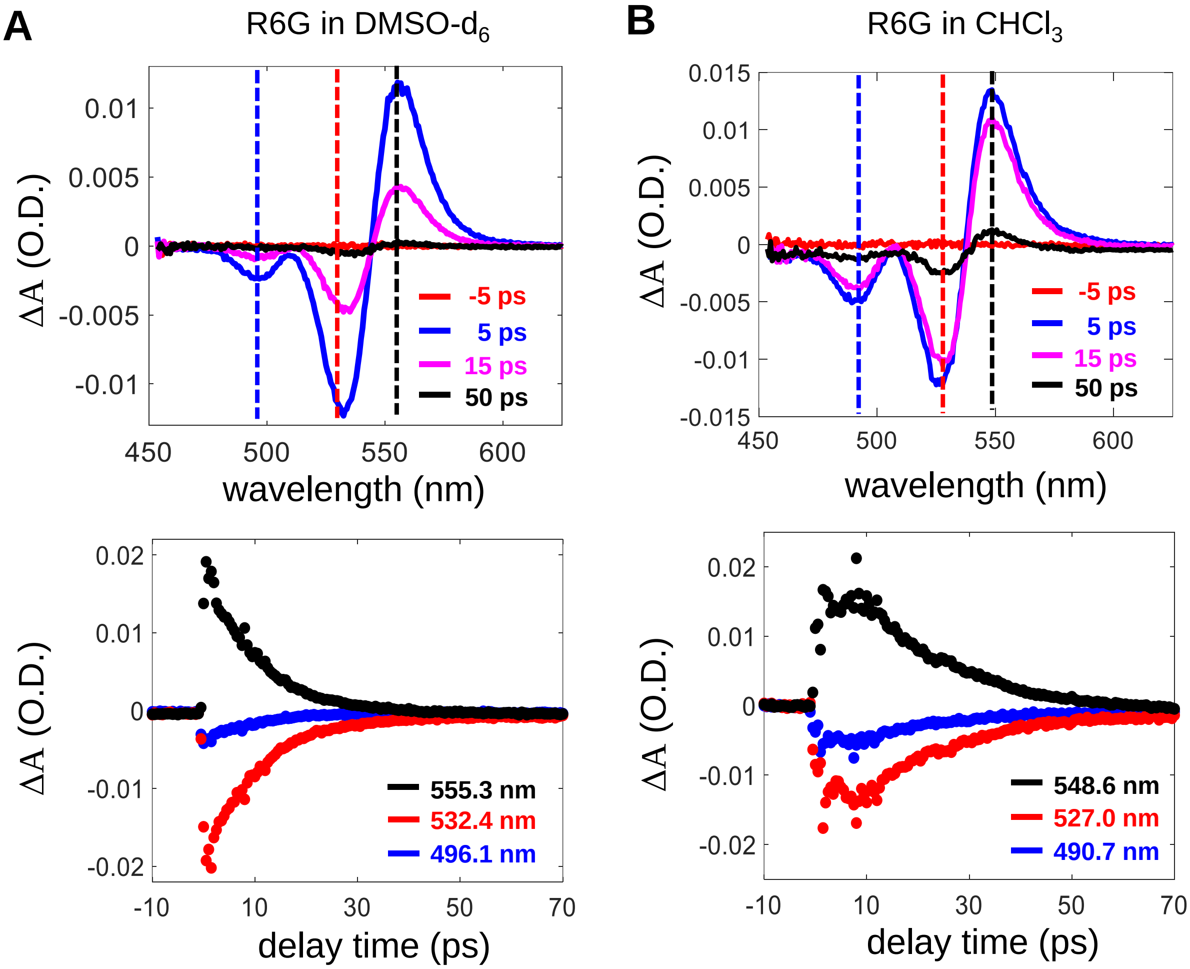

Fig. S1. The TA results of R6G in DMSO-d$_6$ (A) and chloroform (B). The IR pump frequency is 1600 ± 30 cm^{-1}, and the probe is a broadband whitelight pulse. The upper panels show TA spectra at various time delays, and the lower panels show kinetics at several wavelengths that correspond the wavelength positions marked by dashed lines in upper panels.

The R6G solution in chloroform is investigated by measuring its fluorescence intensity following the excitation of a 1600 cm^{-1} IR pulse and then a narrowband visible pulse (520 or 550 nm). The instrument and solution used are the same as the TA experiments above, but the broadband whitelight probe pulse in TA experiments is replaced with a narrowband visible pulse, and the detector receives fluorescence signals rather than the whitelight probe beam. The relative change of fluorescence intensity induced by the IR pulse is measured as 100% × $\left(I_{on} - I_{off}\right)/I_{off}$, where I_{on} or I_{off} are the intensity when the IR beam is unblocked or blocked by a chopper. As shown in Fig. S2, the kinetics of relative fluorescence intensity change show similar patterns to the TA kinetics in Fig. S1B. The IR-induced fluorescence intensity change is attributed to the IR-induced change of electronic absorbance in the visible region. As discussed in the main text, at 550 nm, more absorption of visible photons leads to more fluorescence photons. In contrast, at 520 nm, ground state bleaching leads to reduction of fluorescence signals. The results support the mechanism 1 of MD-WISE imaging of silica beads in the main text, but the change level of fluorescence intensity is small (only a few percentages). This is attributed to the relatively large thickness of the solutions (56 microns) generating defocused background fluorescence signals that is not modulated by the IR pulse.

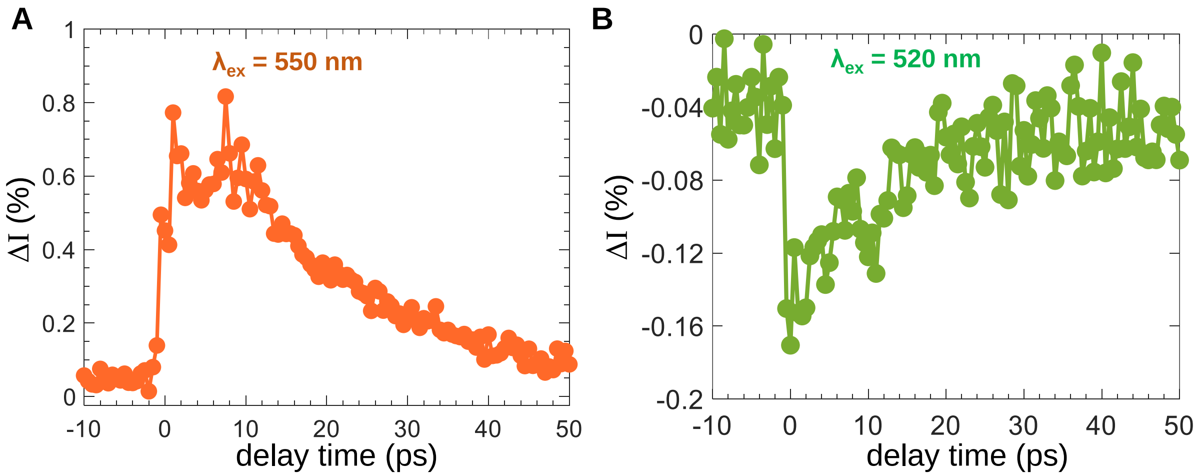

Fig. S2. The transient change of fluorescence intensity of R6G in chloroform. Following the excitation of an IR pulse and then a narrow band visible pulse, the fluorescence signals emitted are collected in the wavelength range of 585 ± 18 nm using a bandpass filter. The IR pump frequency is 1600 ± 30 cm^{-1}, and the visible excitation range is 550 ± 5 nm (A) or 520 ± 5 nm (B).

(b). MD-WISE images of stained silica beads.

In Fig. S3A, MD-WISE images that correspond to the 1600 cm^{-1} kinetic curve in main text Fig. 2E are displayed at a series of delay times. As described in the main text, the kinetic curves are measured by averaging the relative intensity change among all the detector pixels in a 2.5 µm by 2.5 µm box that centers around the microbead. At later delay times, vibrational relaxation and energy redistribution reduces the counts per pixel and the quality of the difference images. The highest quality of difference images is obtained using short delays such as 1 ps. In Fig. S3B, MD-WISE images that correspond to the 1720 cm^{-1} kinetic curve in main text Fig. 2E are

displayed at a series of delay times. Besides the IR frequencies listed in the main text, we also performed MD-WISE imaging using 1650 ± 30 cm^{-1} IR excitation. The IR absorption peak at 1650 cm^{-1} is assigned to another stretch mode of the xanthene ring in R6G. As shown in Fig. S4, the ultrafast decay kinetics are faster than the kinetics measured at 1600 cm^{-1} and 1720 cm^{1}.

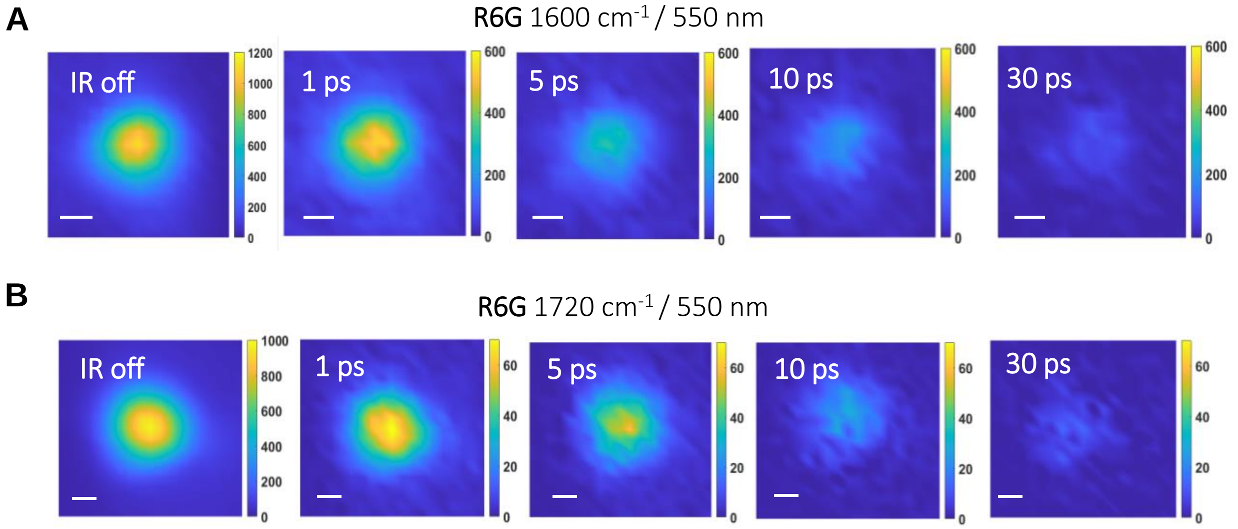

Fig. S3. (A) MD-WISE images obtained using 1600 ± 30 cm^{-1} IR pulse and 550 ± 5 nm visible pulse, and **(B)** MD-WISE images obtained using 1720 ± 30 cm^{-1} IR pulse and 550 ± 5 nm visible pulse. The fluorescence signals are collected in the range of 585 ± 18 nm. Each series in (A) or (B) includes an IR-off image and difference images ($IR_{on} - IR_{off}$) at different delay times. Scale bars are 1 micron in size. The color bars represent CCD counts per pixel.

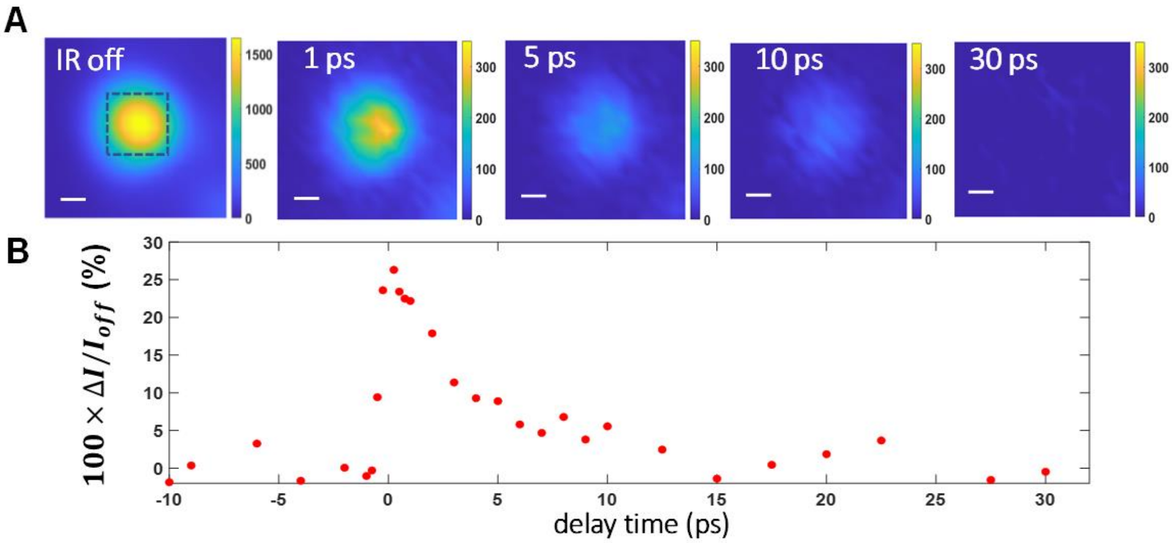

Fig. S4. (A) MD-WISE images obtained using 1650 ± 30 cm^{-1} IR pulse and 550 ± 5 nm visible pulse. The fluorescence signals are collected in the range of 585 ± 18 nm. The series include an IR-off image and difference images ($IR_{on} - IR_{off}$) at different delay times. Scale bars are 1 micron in size. The color bars represent CCD counts per pixel. **(B)** Ultrafast kinetics of fluorescence intensity change at IR excitation of 1650 cm^{-1}. The relative change of counts per pixel is measured using the pixels within the 2.5-micron square box marked by dashed lines in (A).

(c). Kinetics of IR-induced change of fluorescence intensity measured using other substrate materials: azide-functionalized silica and polymethyl methacrylate (PMMA).

Since the R6G dye molecules have overlapping IR spectral features with the silica beads at ~1600 cm^{-1}, we performed MD-WISE experiments on an additional set of substrate materials to investigate whether the IR absorption and the properties of substrate materials have large impacts on the mechanism of ultrafast IR-induced emission intensity change.

One substrate tested is the surface-modified silica microbeads. The surface of silica microbeads contains silanol groups which can be used to anchor organic molecules with distinct vibrational features. Using an established protocol, 3-(azidopropyl)triethoxysilane (Gelest Inc., catalog no. SIA0777.0, structure embedded in Fig. S5A) is grafted onto the surface of silica microbeads (1). In Fig. S5A, the azide-modified silica microbeads (Silica-N_3) show a distinct azide stretch mode at ~2100 cm^{-1} in addition to the absorption features of silica at 1600 cm^{-1} and 1800-2000 cm^{-1}.

Another substrate tested is PMMA microbeads (EpruiBiotech, Shanghai, catalog no. 3-001-3). As shown in Fig. S5B, PMMA lacks vibrational features at ~1600 cm^{-1}, and thus has no overlap with the ring stretch mode of R6G at 1600 cm^{-1}.

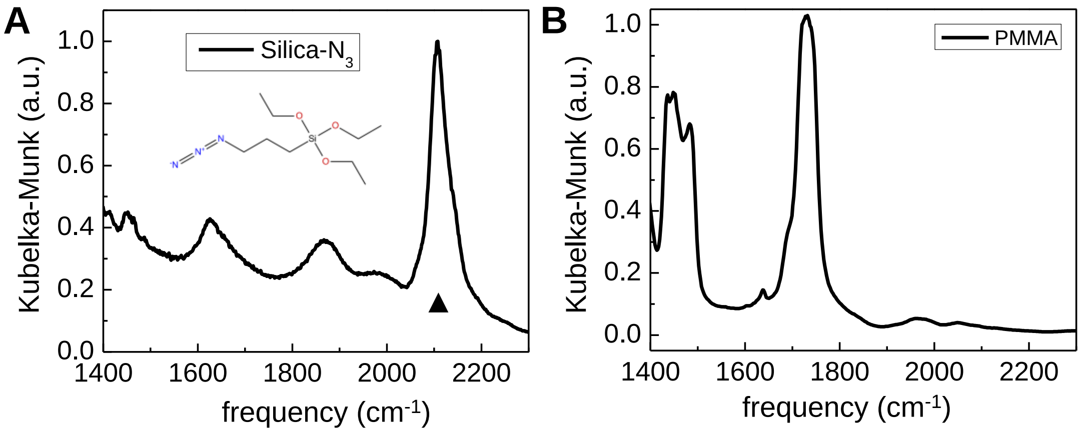

Fig. S5. Diffuse reflectance infrared Fourier transform spectroscopy (DRIFTS) results of azide-modified silica microbeads **(A)** and PMMA microbeads **(B)**. The azide stretch mode is marked by the ▲ symbol in panel A.

The ultrafast kinetics of IR-induced fluorescence intensity change of R6G on three types of substrates are shown in Fig. S6A. The IR frequency is tuned to 1600 ± 30 cm^{-1} for the xanthene ring stretch mode. The results show that, no matter the substrate IR absorption feature overlaps with the R6G feature or not, the kinetics are nearly identical for the three types of substrates. Thus, the IR-induced fluorescence intensity change measured for the silica beads shall be attributed to the excitation of molecular vibrational modes of the adsorbed dyes, rather than the IR absorption of silica.

Furthermore, we test whether exciting the azide stretch mode of Silica-N_3 can transfer energy to R6G modes and cause modulation on fluorescence intensity. As shown in Fig. S6B, there is no modulation on fluorescence intensity using IR frequency at 2100 cm^{-1}. This result agrees well with the flat kinetic traces in main text Fig. 2E using IR frequencies in the range of 1800-2000 cm^{-1}.

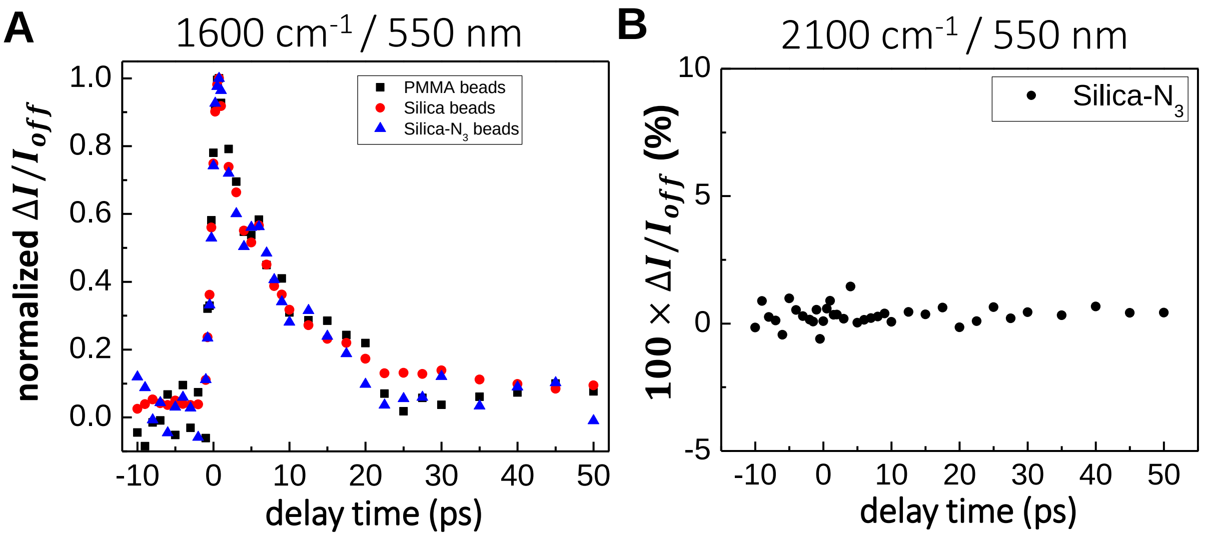

Fig. S6. (A) Normalized ultrafast kinetics of fluorescence intensity change measured with IR excitation of 1600 ± 30 cm^{-1} and visible excitation of 550 ± 5 nm. The results show the three types of substrates stained with R6G have nearly identical kinetics. **(B)** Ultrafast kinetics of fluorescence intensity change of R6G in azide-modified silica microbeads measured with IR excitation of 2100 ± 30 cm^{-1} and visible excitation of 550 ± 5 nm. No IR-induced emission intensity change is observed. The fluorescence signals are collected in the range of 585 ± 18 nm.

III. Additional MD-WISE data on other stained silica beads.

(a). Quantum dots (QDs)

In Fig. S7, MD-WISE images of QD-stained silica beads that correspond to the kinetic curve in main text Fig. 3D are displayed at a series of delay times. For QDs, the IR pulse only has effect on the photoluminescence of QDs when it arrives later than the visible pulse (negative delay times).

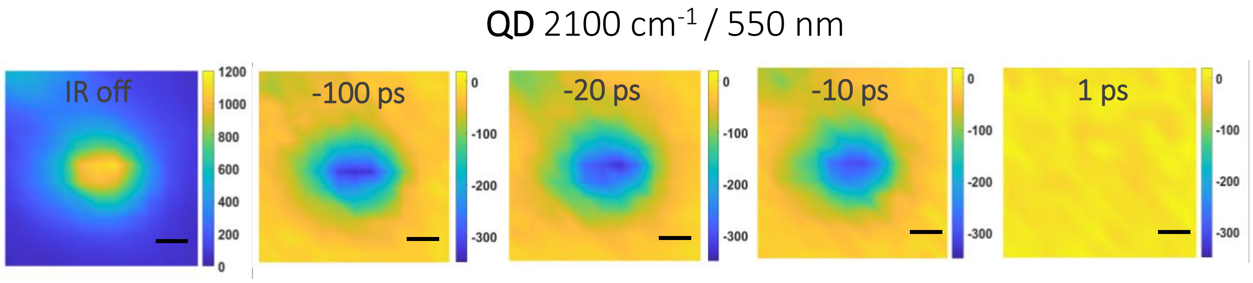

Fig. S7. MD-WISE images of QD stained beads obtained using 2100 ± 30 cm^{-1} IR pulse and 550 ± 5 nm visible pulse. The photoluminescence signals are collected in the range of 585 ± 18 nm. The series include an IR-off image and difference images ($IR_{on} – IR_{off}$) at different delay times. Scale bars are 1 micron in size. The color bars represent CCD counts per pixel.

(b). Fluorescein

In Fig. S8, MD-WISE images of fluorescein-stained silica beads that correspond to the kinetic curve in main text Fig. 4D are displayed at a series of delay times. Only IR frequency of 1600 cm^{-1} that resonantly excites the molecule show effects on fluorescence emission intensity, while the difference images acquired using IR frequency of 2040 cm^{-1} are blank. For 1600 cm^{-1}, similar to the case of R6G, at later delay times, vibrational relaxation and energy redistribution reduces the counts per pixel and the quality of the difference images.

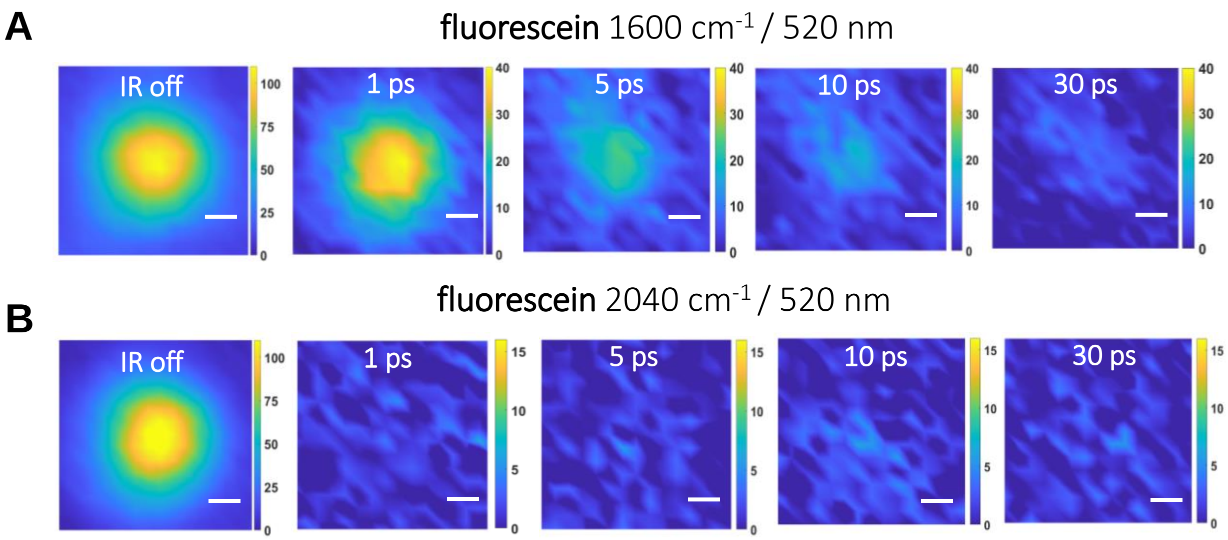

Fig. S8. (A) MD-WISE images of fluorescein-stained beads obtained using 1600 ± 30 cm^{-1} IR pulse and 520 ± 5 nm visible pulse, and **(B)** MD-WISE images of fluorescein-stained beads obtained using 2040 ± 30 cm^{-1} IR pulse and

520 ± 5 nm visible pulse. The fluorescence signals are collected in the range of 585 ± 18 nm. Each series in (A) or (B) include an IR-off image and difference images ($IR_{on} - IR_{off}$) at different delay times. Scale bars are 1 micron in size. The color bars represent CCD counts per pixel.

(c). Fluorescein-5-isothiocyanate (FITC)

Fig. S9, MD-WISE images of FITC-stained silica beads that correspond to the kinetic curve in main text Fig. 4D are displayed at a series of delay times. Both the IR frequencies of 1600 cm^{-1} and 2040 cm^{-1} resonantly excite the molecule and show effects on fluorescence emission intensity. The lifetime of IR-modulation on the fluorescence of FITC is only a few ps.

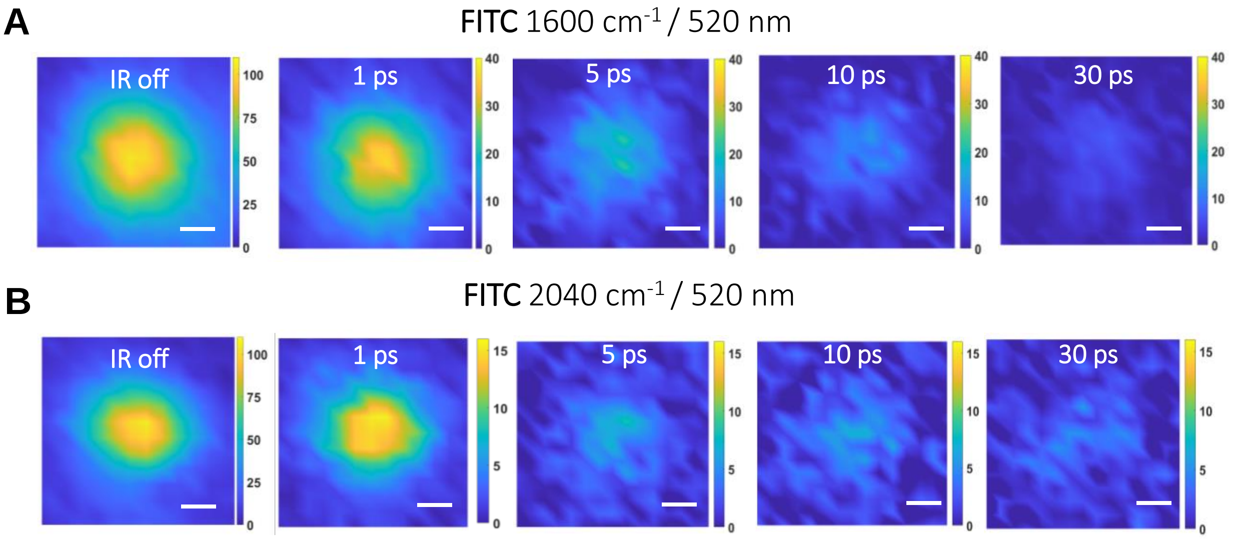

Fig. S9. (A) MD-WISE images of FITC-stained beads obtained using 1600 ± 30 cm^{-1} IR pulse and 520 ± 5 nm visible pulse, and **(B)** MD-WISE images of FITC-stained beads obtained using 2040 ± 30 cm^{-1} IR pulse and 520 ± 5 nm visible pulse. The fluorescence signals are within the range of 585 ± 18 nm. Each series in (A) or (B) include an IR-off image and difference images ($IR_{on} - IR_{off}$) at different delay times. Scale bars are 1 micron in size. The color bars represent CCD counts per pixel.

IV. Additional MD-WISE data on stained cells.

(a). Cells stained with propidium iodide (PI)

In Fig. S10, three replica sets of MD-WISE difference images of PI-stained cancer cells are displayed at different delay times. Since the IR frequency of 2100 cm^{-1} does not excite the vibrational modes of PI dyes, no IR-induced effects are observed. The IR-off images show the stained nucleic acids of the cells, while the difference images at positive or negative delays on show blank.

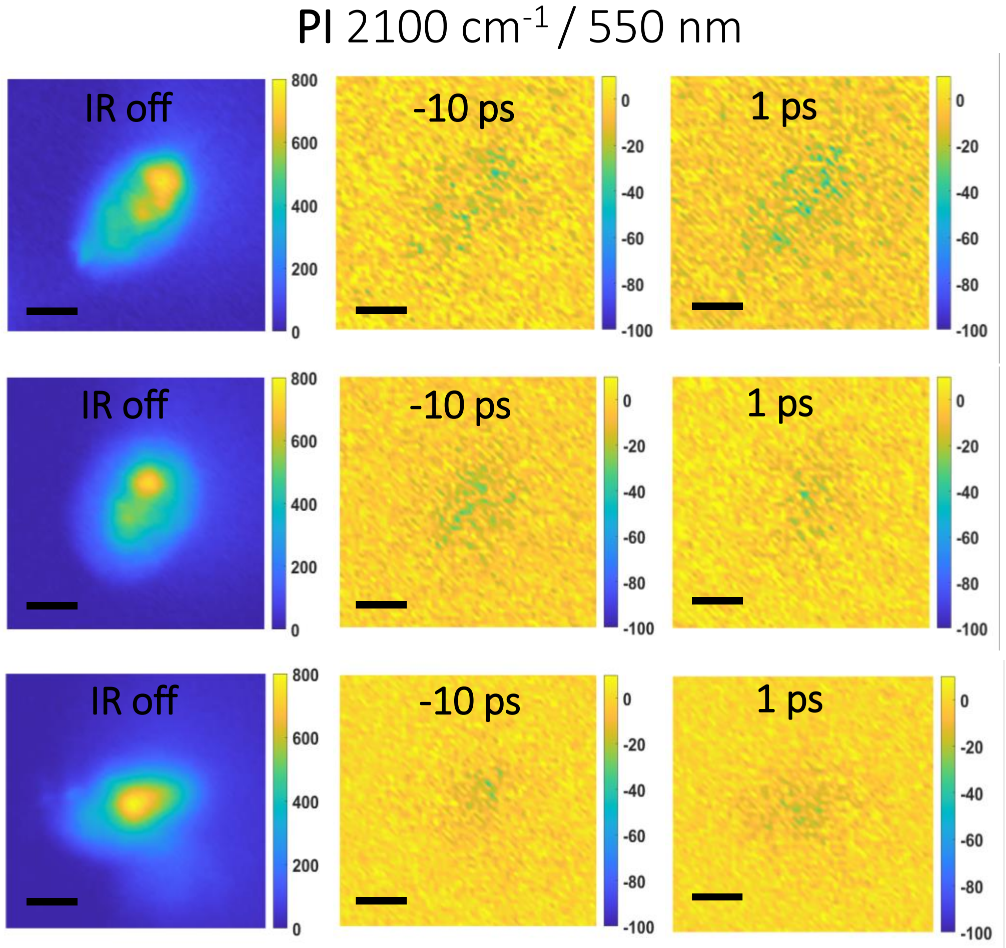

Fig. S10. Three sets of MD-WISE images of PI-stained cells obtained using 2100 ± 30 cm^{-1} IR pulse and 550 ± 5 nm visible pulse. The fluorescence signals are collected in the range of 585 ± 18 nm. The series include IR-off images (left column) and associated difference images ($IR_{on} - IR_{off}$) at -10 ps (middle column) and 1 ps (right column). Scale bars are 5 microns in size. The color bars represent CCD counts per pixel.

(b). Cells stained with streptavidin-coated QD585

In Fig. S11, three replica sets of MD-WISE difference images of QD-stained cancer cells are displayed at different delay times. The IR-off images show the stained cell membranes. The difference images at the negative delay of -10 ps reproduce the outline of cell membranes. The

difference images at the positive delay of 1 ps show blank, since the IR pulse can only modulate the photoluminescence intensity of QDs when it arrives later than visible pulse (Fig. S12).

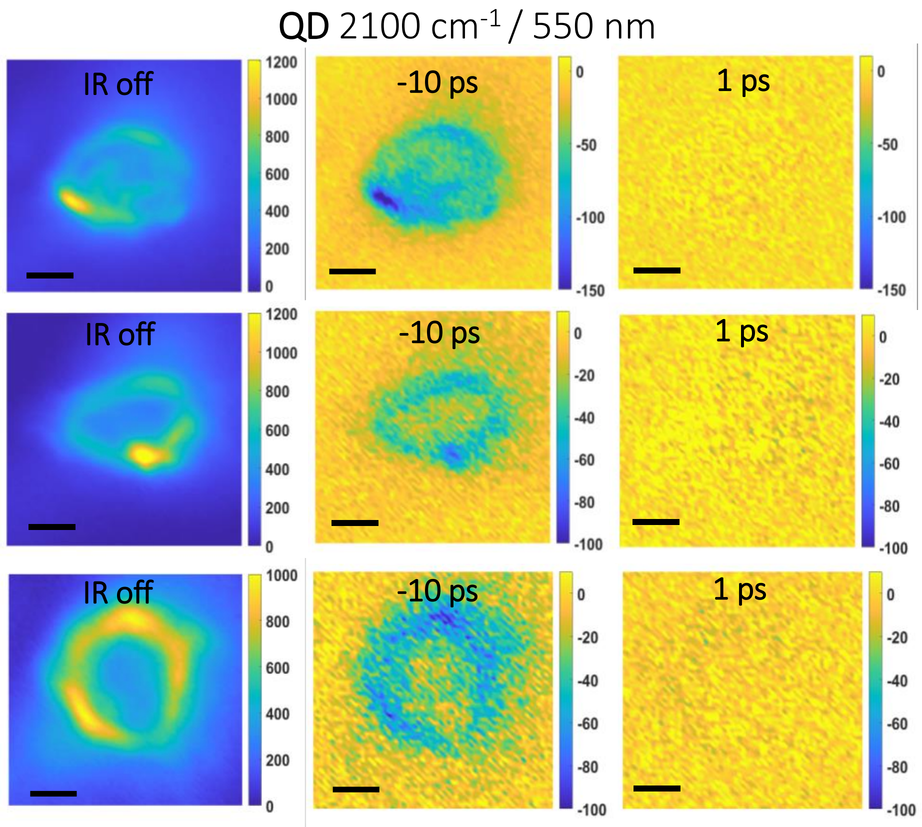

Fig. S11. Three sets of MD-WISE images of QD-stained cells obtained using 2100 ± 30 cm^{-1} IR pulse and 550 ± 5 nm visible pulse. The photoluminescence signals are collected in the range of 585 ± 18 nm. The series include IR-off images (left column) and associated difference images ($IR_{on} - IR_{off}$) at -10 ps (middle column) and 1 ps (right column). Scale bars are 5 microns in size. The color bars represent CCD counts per pixel.

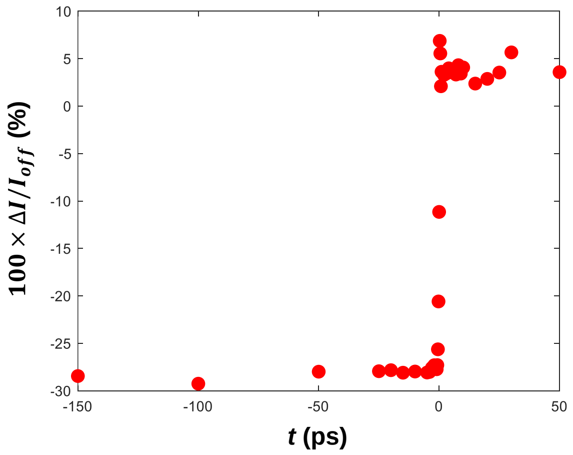

Fig. S12. Ultrafast kinetics of IR-induced photoluminescence intensity change of QD-stained cells obtained using 2100 ± 30 cm^{-1} IR pulse and 550 ± 5 nm visible pulse. The photoluminescence signals are collected in the range of 585 ± 18 nm.

V. Quantum chemical vibrational analysis of R6G dye

(a). Overview and methods

Gaussian 09, Revision D (2) was used to perform quantum chemical calculations for identifying and analyzing the vibrational modes, vibrational frequencies, and coupling between functional groups in the molecule. The density functional theory (3) calculations were carried out using RB3LYP/6-311++G(d,p) basis sets (4). The structure of R6G molecular cation was optimized in vacuo using a crystal structure as the initial guess (5, 6). Then vibrational frequency analysis was performed. The optimized results of R6G cation in vacuum was then used as the initial structure for the vibrational frequency analysis of R6G cations with polarizable continuum models in three different implicit solvent environments: water, methanol, and acetone. The optimized structure of R6G cation in vacuum and the numbering of atoms are displayed as an example in Fig. S13. The atomic coordinates of R6G cations in different environments are listed in Table S1-S2. The frequency and intensity of all the modes above 1000 cm^{-1} are listed in Table S3.

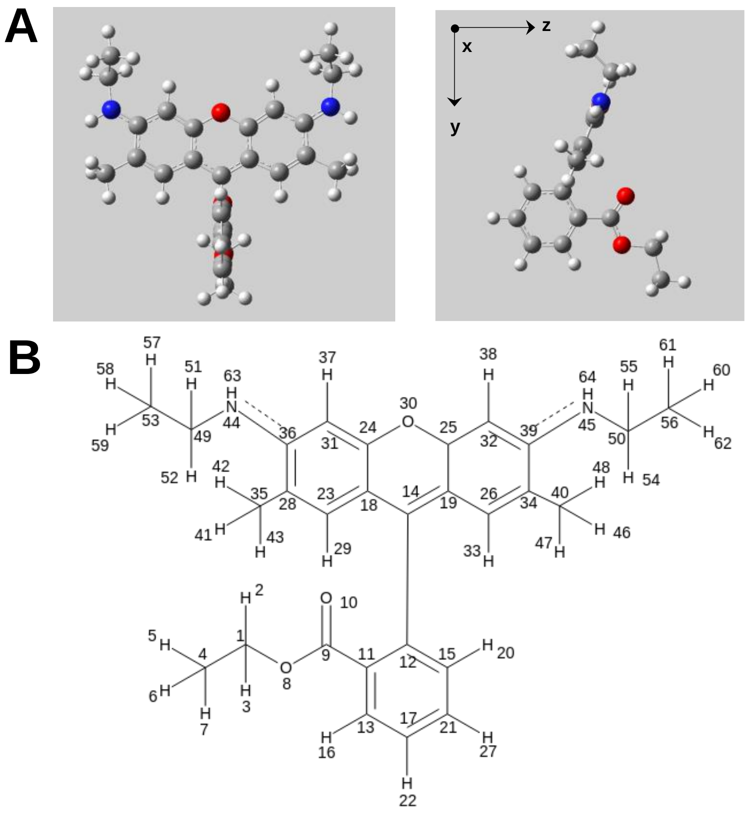

Fig. S13. (A) Optimized structure of R6G cation in vacuum, viewed from two different angles. **(B)** Numbering of atoms in the R6G cation.

		vacuum			water		
**atom number**	**element**	**X**	**Y**	**Z**	**X**	**Y**	**Z**
1	C	-0.001279	4.172279	2.934740	-0.001689	4.318494	2.855955
2	H	0.882432	3.642945	3.296271	0.883701	3.811077	3.242948
3	H	-0.884687	3.642360	3.296154	-0.887125	3.811002	3.242755
4	C	-0.001787	5.631469	3.335211	-0.001782	5.794832	3.188760
5	H	0.885291	6.144704	2.958212	0.886493	6.288716	2.788801
6	H	-0.001885	5.707791	4.425403	-0.001909	5.917944	4.274569
7	H	-0.889154	6.144118	2.958094	-0.889999	6.288658	2.788602
8	O	-0.001164	4.114239	1.477968	-0.001539	4.194327	1.404511
9	C	-0.000724	2.896884	0.921870	-0.001118	2.954435	0.898202
10	O	-0.000439	1.865500	1.560108	-0.000840	1.953544	1.587765
11	C	-0.000647	2.948480	-0.574196	-0.001089	2.944475	-0.596520
12	C	-0.000274	1.750584	-1.317483	-0.000633	1.723128	-1.301116
13	C	-0.000934	4.176377	-1.248266	-0.001534	4.150125	-1.311919
14	C	0.000036	0.392375	-0.690643	-0.000146	0.378180	-0.646203
15	C	-0.000194	1.816738	-2.714362	-0.000642	1.745476	-2.699636
16	H	-0.001219	5.088701	-0.667505	-0.001884	5.083050	-0.765451
17	C	-0.000853	4.227689	-2.637481	-0.001535	4.157224	-2.701553
18	C	1.214032	-0.276148	-0.440157	1.214141	-0.285342	-0.388885
19	C	-1.213658	-0.276689	-0.440143	-1.213967	-0.286171	-0.388804
20	H	0.000098	0.897489	-3.288985	-0.000298	0.808257	-3.243658
21	C	-0.000480	3.044835	-3.372106	-0.001088	2.950727	-3.397386
22	H	-0.001079	5.185740	-3.143339	-0.001884	5.098843	-3.237279
23	C	2.503249	0.279132	-0.680815	2.503503	0.269595	-0.635000
24	C	1.182443	-1.583295	0.110501	1.184040	-1.592977	0.163061
25	C	-1.181489	-1.583806	0.110555	-1.182955	-1.593796	0.163096
26	C	-2.503128	0.277994	-0.680815	-2.503715	0.267922	-0.634750
27	H	-0.000411	3.073215	-4.455534	-0.001087	2.942725	-4.481023
28	C	3.665432	-0.388633	-0.405469	3.666385	-0.400173	-0.367675
29	H	2.558102	1.276220	-1.099995	2.559617	1.267231	-1.052723
30	O	0.000622	-2.197488	0.375026	0.000759	-2.205493	0.432888
31	C	2.328816	-2.294119	0.400959	2.329858	-2.305777	0.445735
32	C	-2.327536	-2.295136	0.401049	-2.328277	-2.307419	0.445719
33	H	-2.558436	1.275042	-1.100030	-2.560537	1.265568	-1.052346
34	C	-3.665015	-0.390271	-0.405419	-3.666133	-0.402629	-0.367370
35	C	5.007430	0.240989	-0.673530	5.006605	0.228853	-0.643329
36	C	3.588948	-1.717493	0.158780	3.591580	-1.732834	0.190826
37	H	2.228387	-3.288173	0.811758	2.231834	-3.301239	0.853881
38	H	-2.226645	-3.289160	0.811801	-2.229546	-3.302846	0.853778
39	C	-3.587948	-1.719086	0.158904	-3.590407	-1.735353	0.190854
40	C	-5.007274	0.238817	-0.673455	-5.006763	0.225723	-0.642548
41	H	5.597825	0.344837	0.243961	5.606457	0.318588	0.268980
42	H	5.598374	-0.345946	-1.386085	5.588553	-0.355705	-1.364471
43	H	4.887238	1.238060	-1.096910	4.880567	1.229664	-1.055954
44	N	4.737196	-2.371346	0.452894	4.737367	-2.393119	0.465071
45	N	-4.735958	-2.373275	0.453320	-4.735713	-2.396553	0.464945
46	H	-5.597756	0.342273	0.244019	-5.606363	0.314915	0.269978
47	H	-4.887462	1.235987	-1.096702	-4.881366	1.226706	-1.054957
48	H	-5.597951	-0.348223	-1.386162	-5.588626	-0.358945	-1.363663
49	C	4.850366	-3.723048	1.002418	4.848362	-3.757453	0.981106
50	C	-4.848652	-3.725298	1.002105	-4.845623	-3.761083	0.980705
51	H	4.095162	-3.848512	1.782466	4.103157	-3.900350	1.767059
52	H	5.819107	-3.776896	1.503169	5.825611	-3.829847	1.460868
53	C	4.740397	-4.830267	-0.049721	4.713548	-4.834333	-0.099425
54	H	-5.816980	-3.779478	1.503618	-5.822892	-3.834419	1.460285
55	H	-4.092815	-3.851043	1.781463	-4.100430	-3.903469	1.766762
56	C	-4.739325	-4.831887	-0.050745	-4.709669	-4.837666	-0.099983
57	H	3.778836	-4.800400	-0.566796	3.741684	-4.785105	-0.595454
58	H	4.839441	-5.808127	0.428123	4.816059	-5.824495	0.351954
59	H	5.531066	-4.737605	-0.797873	5.492238	-4.720580	-0.857326
60	H	-4.837989	-5.810034	0.426595	-4.811408	-5.827990	0.351216
61	H	-3.778097	-4.801620	-0.568416	-3.737753	-4.787516	-0.595818
62	H	-5.530503	-4.738884	-0.798324	-5.488313	-4.724468	-0.858013
63	H	5.600465	-1.907533	0.220308	5.602869	-1.940107	0.216526
64	H	-5.599285	-1.910306	0.219209	-5.601567	-1.944287	0.216244

Table S1. Atomic coordinates of R6G cation in vacuum and water.

		methanol			acetone		
atom number	element	X	Y	Z	X	Y	Z
1	C	0.000416	4.318476	2.854948	0.000901	4.318985	2.853820
2	H	0.885408	3.810260	3.241826	0.885984	3.810768	3.240485
3	H	-0.885251	3.811323	3.241690	-0.884570	3.811452	3.240504
4	C	0.001285	5.794756	3.188025	0.001465	5.795225	3.186912
5	H	0.889822	6.288187	2.788108	0.889827	6.288936	2.786955
6	H	0.001270	5.917773	4.273848	0.001485	5.918330	4.272731
7	H	-0.886580	6.289281	2.787964	-0.886492	6.289625	2.786910
8	O	0.000431	4.194525	1.403500	0.000825	4.195045	1.402285
9	C	0.000198	2.954528	0.897320	0.000615	2.954895	0.896422
10	O	0.000073	1.953617	1.586601	0.000415	1.954022	1.585536
11	C	0.000140	2.944486	-0.597477	0.000498	2.944557	-0.598473
12	C	0.000014	1.722879	-1.301513	0.000234	1.722544	-1.301733
13	C	0.000184	4.149848	-1.313313	0.000652	4.149491	-1.314906
14	C	-0.000050	0.378236	-0.645956	0.000025	0.378374	-0.645261
15	C	-0.000075	1.744607	-2.700057	0.000151	1.743441	-2.700265
16	H	0.000279	5.082918	-0.767078	0.000853	5.082829	-0.769127
17	C	0.000097	4.156347	-2.702962	0.000557	4.155171	-2.704610
18	C	1.213955	-0.285816	-0.388624	1.213967	-0.285783	-0.387765
19	C	-1.214139	-0.285621	-0.388486	-1.214138	-0.285344	-0.387664
20	H	-0.000178	0.807129	-3.243659	-0.000047	0.805638	-3.243332
21	C	-0.000033	2.949569	-3.398309	0.000309	2.948012	-3.399252
22	H	0.000127	5.097722	-3.239113	0.000681	5.096220	-3.241321
23	C	2.503519	0.269051	-0.633677	2.503557	0.269373	-0.631950
24	C	1.183263	-1.593686	0.162685	1.183070	-1.593893	0.162862
25	C	-1.183622	-1.593511	0.162758	-1.183671	-1.593478	0.162928
26	C	-2.503631	0.269515	-0.633238	-2.503544	0.270285	-0.631738
27	H	-0.000106	2.941173	-4.481947	0.000238	2.938973	-4.482888
28	C	3.666033	-0.401199	-0.366088	3.665928	-0.401097	-0.364338
29	H	2.560018	1.267186	-1.050229	2.560121	1.268101	-1.047124
30	O	-0.000216	-2.206012	0.431737	-0.000400	-2.206389	0.431001
31	C	2.328794	-2.307163	0.445036	2.328561	-2.307765	0.444728
32	C	-2.329249	-2.306858	0.445065	-2.329395	-2.306969	0.444813
33	H	-2.560015	1.267728	-1.049602	-2.559786	1.269045	-1.046881
34	C	-3.666240	-0.400514	-0.365503	-3.666136	-0.399785	-0.364074
35	C	5.006735	0.227957	-0.638961	5.006823	0.228710	-0.634546
36	C	3.590622	-1.734391	0.190886	3.590291	-1.734894	0.191052
37	H	2.230290	-3.302836	0.852561	2.229830	-3.303746	0.851437
38	H	-2.230854	-3.302595	0.852454	-2.230995	-3.303009	0.851464
39	C	-3.591018	-1.733897	0.191028	-3.590935	-1.733644	0.191223
40	C	-5.006821	0.229255	-0.637528	-5.006824	0.230525	-0.634144
41	H	5.604716	0.317070	0.274676	5.603040	0.317363	0.280321
42	H	5.590119	-0.356030	-1.359416	5.591755	-0.354420	-1.354432
43	H	4.881661	1.229047	-1.051140	4.882154	1.230034	-1.046209
44	N	4.736418	-2.395140	0.464487	4.736338	-2.395859	0.463857
45	N	-4.736879	-2.394650	0.464405	-4.737198	-2.394245	0.463998
46	H	-5.604232	0.318196	0.276495	-5.602974	0.319249	0.280759
47	H	-4.881546	1.230489	-1.049309	-4.881817	1.231865	-1.045664
48	H	-5.590841	-0.354142	-1.357935	-5.592000	-0.352284	-1.354092
49	C	4.847457	-3.759570	0.980240	4.847644	-3.760409	0.979092
50	C	-4.847938	-3.759224	0.979794	-4.848950	-3.758819	0.979072
51	H	4.102389	-3.902417	1.766345	4.102731	-3.903375	1.765327
52	H	5.824736	-3.832134	1.460010	5.824946	-3.833131	1.458927
53	C	4.712437	-4.836415	-0.100295	4.712593	-4.837140	-0.101527
54	H	-5.825330	-3.831983	1.459308	-5.826292	-3.831301	1.458863
55	H	-4.103048	-3.902192	1.766046	-4.104111	-3.902094	1.765318
56	C	-4.712561	-4.835833	-0.100937	-4.714179	-4.835473	-0.101656
57	H	3.740576	-4.787092	-0.596313	3.740825	-4.787698	-0.597720
58	H	4.814917	-5.826623	0.350965	4.814939	-5.827457	0.349524
59	H	5.491009	-4.722872	-0.858341	5.491177	-4.723769	-0.859596
60	H	-4.815069	-5.826139	0.350105	-4.816856	-5.825804	0.349288
61	H	-3.740584	-4.786337	-0.596709	-3.742372	-4.786285	-0.597800
62	H	-5.490959	-4.722207	-0.859147	-5.492692	-4.721783	-0.859753
63	H	5.601712	-1.941831	0.215851	5.601252	-1.942716	0.213652
64	H	-5.602168	-1.941422	0.215574	-5.601954	-1.940817	0.213768

Table S2. Atomic coordinates of R6G cation in methanol and acetone.

	frequency (cm^{-1})				IR intensity (km/mol)			
	**vacuum**	**water**	**methanol**	**acetone**	**vacuum**	**water**	**methanol**	**acetone**
83	1021.28	1021.09	1021.01	1020.93	0.1107	0.3837	0.3591	0.3604
84	1023.51	1023.73	1023.87	1023.95	1.4800	68.5142	67.7481	67.2432
85	1027.03	1025.96	1025.70	1025.52	46.4093	2.7179	2.7042	2.6796
86	1033.69	1033.78	1033.63	1033.55	96.7390	329.1795	315.3931	302.5882
87	1056.17	1054.25	1054.30	1054.41	4.7702	6.3961	6.1829	5.9760
88	1061.77	1059.26	1058.92	1058.74	1.0243	7.2357	6.5911	6.7210
89	1061.84	1059.33	1059.00	1058.80	0.0976	0.2116	0.7139	0.4025
90	1066.47	1065.86	1066.00	1066.12	5.3765	15.3165	14.7875	14.2829
91	1069.72	1068.48	1068.61	1068.78	6.0784	8.7832	9.3635	9.5410
92	1098.42	1095.77	1095.88	1095.99	86.4319	171.0064	167.5217	165.0365
93	1105.33	1102.80	1102.86	1102.94	107.2901	149.3434	150.1146	149.6237
94	1106.62	1104.84	1104.84	1104.91	61.5949	130.6691	129.0868	127.4667
95	1133.00	1130.77	1130.83	1130.91	8.2679	19.4849	18.8563	18.2490
96	1147.50	1146.01	1146.06	1146.20	6.3007	12.0263	11.4577	10.9895
97	1153.72	1152.93	1152.75	1152.67	7.7319	19.3738	18.6133	18.0801
98	1160.21	1158.44	1158.34	1158.30	17.1638	22.4030	22.7379	23.0342
99	1169.21	1163.17	1163.57	1163.93	111.8170	190.2419	188.4449	186.4874
100	1174.95	1172.25	1172.36	1172.46	2.3510	6.7185	6.3488	6.0155
101	1183.25	1182.76	1182.81	1182.90	0.8692	17.8338	16.6004	15.3824
102	1192.64	1186.16	1187.06	1187.87	3.1676	2.9890	2.7482	2.5467
103	1199.31	1197.20	1197.20	1197.25	1.5390	0.6411	0.6866	0.7389
104	1215.12	1211.11	1211.17	1211.31	44.3007	155.5757	150.7172	146.3949
105	1215.58	1215.18	1215.20	1215.29	10.4185	12.1061	12.0395	12.0482
106	1264.07	1263.29	1263.22	1263.18	178.5692	762.0616	728.2297	695.6885
107	1283.52	1267.82	1268.32	1268.80	433.2214	859.0036	850.2553	842.0037
108	1287.33	1288.60	1288.19	1287.82	192.8846	28.3065	28.1195	27.9779
109	1290.40	1292.39	1292.28	1292.24	24.3212	220.0824	193.9639	170.8829
110	1293.22	1292.51	1292.68	1292.85	1.3848	1.9761	1.9411	1.9733
111	1298.04	1297.41	1297.43	1297.41	2.3084	1.9346	1.9747	2.0505
112	1316.99	1316.58	1316.53	1316.51	15.7910	39.8318	36.4974	33.9324
113	1326.42	1321.32	1321.63	1321.91	37.8761	2756.0700	2588.4144	2418.7514
114	1326.91	1325.45	1325.52	1325.55	192.5227	40.9546	41.5717	41.7772
115	1332.56	1331.42	1331.45	1331.57	26.3837	916.5779	995.6621	1075.5075
116	1340.64	1332.36	1332.41	1332.45	1194.3390	43.2193	42.9687	43.3080
117	1378.58	1377.81	1377.75	1377.71	1.6851	14.8449	13.9555	12.6827
118	1386.92	1385.52	1385.50	1385.54	92.0089	141.8075	140.5607	139.4684
119	1391.95	1391.19	1391.14	1391.13	13.1959	61.4256	59.0184	56.2183
120	1400.50	1397.27	1397.37	1397.45	9.4751	14.9330	14.6609	14.4209
121	1400.61	1400.04	1399.82	1399.65	16.4978	29.5787	27.1188	25.5360
122	1416.52	1406.33	1406.72	1407.15	16.1624	16.6268	16.8015	16.7333
123	1416.61	1406.49	1406.88	1407.30	2.6363	0.6732	0.4422	0.4295

124	1417.76	1411.10	1410.73	1410.50	4.5214	0.8061	0.8830	0.9753
125	1419.15	1411.67	1411.42	1411.29	3.3223	31.3087	33.4780	34.9959
126	1428.70	1419.32	1419.63	1419.94	10.3034	7.8359	8.0132	8.1780
127	1447.58	1446.49	1446.62	1446.74	120.5303	185.4730	183.5106	181.0806
128	1460.60	1456.18	1456.30	1456.50	67.3700	126.3559	125.7848	125.4190
129	1472.07	1469.39	1469.60	1469.81	9.1187	13.8344	13.4485	13.1429
130	1487.35	1476.32	1476.63	1476.94	10.1184	11.3116	11.2879	11.1815
131	1487.66	1478.26	1477.90	1477.61	45.2969	7.6466	11.7437	14.7008
132	1488.37	1478.43	1478.22	1478.08	16.3386	24.6107	23.2644	22.1559
133	1488.59	1479.57	1479.91	1480.26	16.2072	124.6956	119.6583	114.3595
134	1491.86	1480.90	1481.30	1481.71	1.5466	2.0254	2.0021	1.9621
135	1494.27	1484.11	1484.62	1484.94	2.0071	3.0014	3.0444	14.6129
136	1495.67	1484.39	1484.65	1485.12	32.9523	21.4591	16.8627	3.0684
137	1496.23	1485.17	1485.49	1485.83	0.4137	0.0562	0.1500	0.2821
138	1498.16	1485.98	1486.32	1486.73	3.6304	313.7868	294.6754	275.8538
139	1506.89	1506.92	1506.89	1506.90	68.8235	13.5348	13.1793	12.6232
140	1510.70	1507.49	1507.54	1507.62	5.1891	159.1702	115.1593	75.9495
141	1512.61	1509.49	1509.27	1509.17	13.7976	274.3679	317.2930	363.8552
142	1512.77	1509.95	1510.14	1510.30	20.4101	14.0279	8.8884	6.3681
143	1515.64	1511.15	1511.11	1511.24	13.3810	51.4473	57.5503	61.7442
144	1517.88	1515.77	1515.94	1516.10	3.5751	6.6438	6.4050	6.1635
145	1530.68	1521.70	1522.14	1522.59	749.9864	1813.4693	1755.2090	1689.9236
146	1535.34	1535.24	1535.31	1535.35	32.5002	35.3201	33.0173	30.8236
147	1560.60	1551.76	1552.16	1552.58	369.5476	629.6269	618.4447	606.0617
148	1585.12	1577.20	1577.65	1578.06	73.4126	62.6507	60.4359	58.6146
149	1591.13	1589.46	1589.98	1590.48	382.4680	705.3651	698.7154	695.8972
150	1599.41	1596.53	1597.01	1597.54	199.1142	420.6167	412.9426	404.3088
151	1607.22	1605.15	1605.31	1605.50	18.7643	43.0498	41.8391	41.0375
152	1632.71	1631.62	1631.73	1631.85	4.9485	9.4990	9.2504	8.9941
153	1647.36	1635.92	1636.29	1636.64	1102.9195	1559.8124	1546.9814	1530.9197
154	1685.44	1679.75	1679.96	1680.13	313.4626	454.1640	448.9968	444.3885
155	1750.42	1724.25	1725.57	1726.77	290.5695	590.2452	574.1302	559.0726
156	3011.45	3014.36	3013.95	3013.79	50.3714	119.2416	112.1614	117.5624
157	3011.52	3014.50	3014.10	3013.93	1.9947	3.5958	8.6041	1.0311
158	3036.58	3034.28	3034.36	3034.36	6.5181	13.4671	13.5437	13.5433
159	3036.64	3034.34	3034.42	3034.42	23.7765	59.2489	57.9520	56.7628
160	3040.62	3038.58	3038.63	3038.67	12.6904	23.1803	22.9175	22.6623
161	3052.12	3056.76	3056.55	3056.39	19.4577	36.8685	35.9709	35.6592
162	3052.32	3056.91	3056.70	3056.54	32.6185	79.7855	77.8404	75.5132
163	3056.34	3059.32	3058.93	3058.83	7.0400	21.0028	20.0922	6.2687
164	3056.37	3059.38	3059.02	3058.84	12.4806	20.5580	19.7503	31.9950
165	3059.34	3060.71	3060.73	3060.83	10.5225	25.1701	24.2406	23.3833
166	3086.44	3091.22	3090.90	3090.51	1.1662	0.2783	0.1272	0.3109

167	3086.48	3091.24	3090.91	3090.53	1.0074	0.3129	0.5053	0.3665
168	3095.72	3096.36	3096.37	3096.42	0.3928	0.0001	0.0000	0.0023
169	3106.30	3102.21	3102.36	3102.43	3.7640	5.5069	5.0819	4.9197
170	3106.37	3102.23	3102.39	3102.46	42.7852	103.8178	101.2658	98.5869
171	3108.56	3106.74	3106.78	3106.79	16.1957	35.3121	34.4627	33.6632
172	3113.05	3113.20	3113.02	3112.78	62.1423	114.0702	124.6808	118.0493
173	3113.11	3113.21	3113.03	3112.81	2.2874	15.4672	2.2248	6.3626
174	3119.29	3117.91	3117.50	3117.28	2.2645	0.5773	10.9407	6.7637
175	3119.33	3117.93	3117.55	3117.31	10.4675	33.5144	22.5507	26.0807
176	3121.12	3118.81	3118.89	3119.01	26.1606	64.1916	61.6110	59.2858
177	3173.57	3178.34	3178.12	3177.93	0.8376	0.3395	0.3564	0.3757
178	3184.78	3187.86	3187.70	3187.57	3.3905	9.9007	9.7103	9.5461
179	3191.21	3192.30	3191.72	3191.21	5.0961	14.2380	13.3254	12.4831
180	3191.56	3192.69	3192.10	3191.59	2.8767	7.6899	7.2259	6.7893
181	3196.05	3198.53	3198.38	3198.27	4.9218	17.0230	16.4782	15.9871
182	3217.03	3217.66	3217.55	3217.51	1.1414	4.0956	3.9981	3.8842
183	3221.91	3222.35	3222.33	3222.35	2.0408	4.0193	3.9452	3.8750
184	3222.20	3222.60	3222.59	3222.60	0.3415	0.8806	0.8314	0.7900
185	3640.97	3631.67	3631.21	3630.96	303.7574	653.6930	640.2003	628.7302
186	3641.57	3632.47	3632.01	3631.75	7.6687	0.6127	1.9487	0.5578

Table S3. Calculated vibrational frequencies and infrared absorption intensities of R6G vibrational modes. Vibrational modes related to MD-WISE experiments are highlighted by yellow.

(b). Calculated IR absorption spectra

The calculated vibrational IR spectra of R6G cations in different environments are shown in Fig. S14. The spectra are similar for R6G cations in water, methanol and acetone, which are redshifted from the spectrum in vacuum. The calculated spectra largely match the FTIR pattern of R6G shown in main text Fig. 2A, though the exact center positions of the peaks differ slightly from the experimental results. Three calculated xanthene ring modes (number 149, 150, 153 in Table S3) related to MD-WISE imaging are marked by the hexagon signs in Fig. S14. The stretch mode (number 155 in Table S3) of the ester group is marked by the triangle signs. Each of these four modes is analyzed with details below using water solvent as the example, since different solvents only yield negligible results. Vibrational modes with negligible IR absorption intensities or modes outside of the frequency ranges used in MD-WISE imaging are not subject to further analysis here.

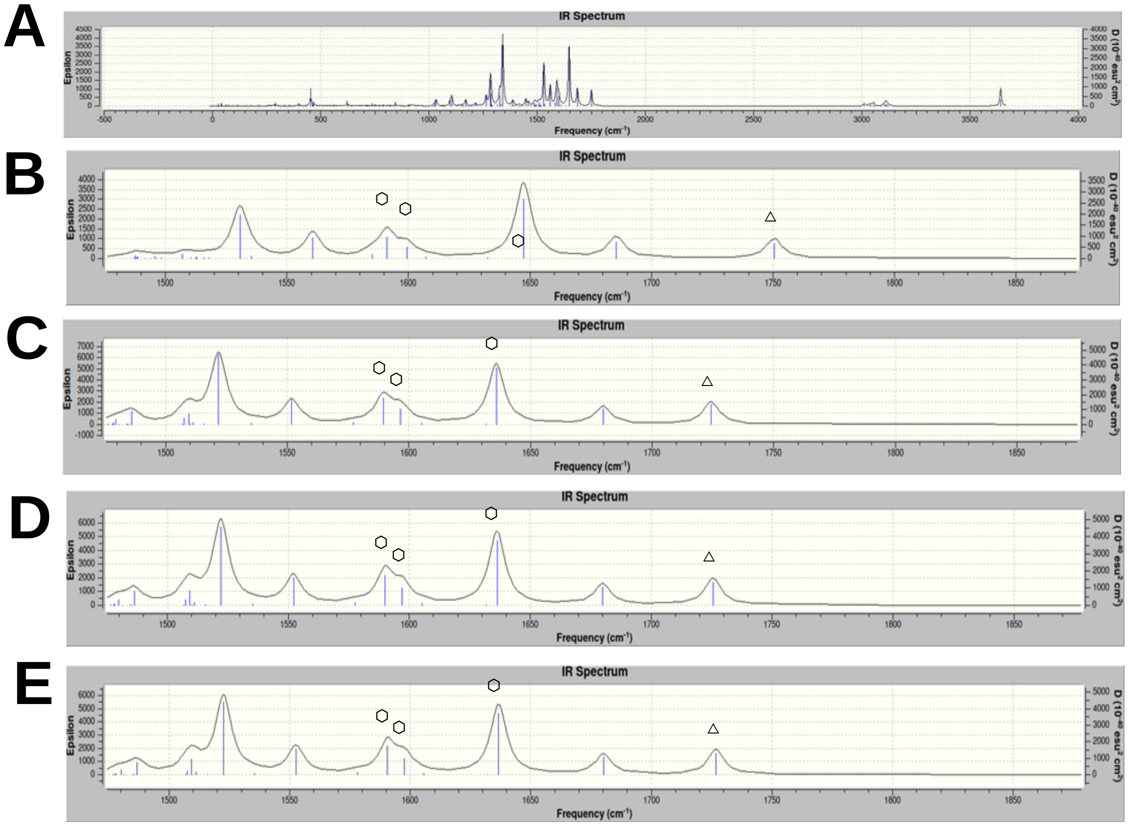

Fig. S14. IR spectra of R6G cations in various environments. **(A)** full IR spectrum in vacuum, **(B-E)** IR spectra in the range between 1475-1875 cm^{-1} for vacuum (B), water (C), methanol (D), and acetone (E). △ marks the peak of ester and ⬡ marks the peaks of xanthene rings, which largely match the peaks observed in FTIR experiments.

(c). Analysis of mode 149 and 150 (xanthene ring modes)

The calculated vibrational modes 149-150 in water at ~1600 cm^{-1} largely only involve the xanthene ring atoms, with minor coupling to the displacements of atoms in the phenyl ring attached to the xanthene ring. The atoms involved and the displacements of each atom are shown in Fig. S15 and Table S4-S5.

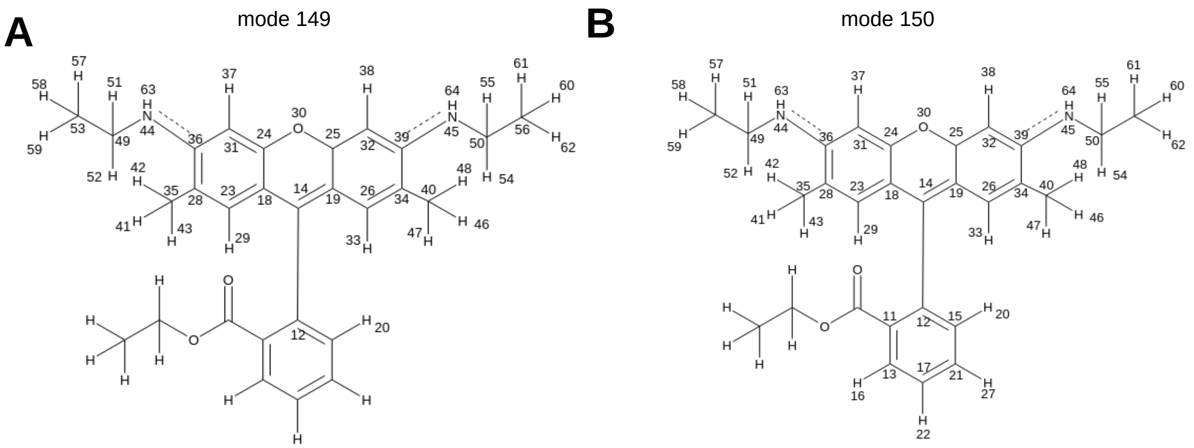

Fig. S15. Atoms involved in mode 149 **(A)** and mode 150 **(B)**.

mode 149				
atom number	element	X	Y	Z
12	C	-0.01	0.00	0.00
14	C	0.11	0.00	0.00
18	C	-0.09	0.01	0.00
19	C	-0.09	-0.01	0.00
20	H	-0.01	0.00	0.00
23	C	0.03	0.01	-0.01
24	C	-0.01	0.03	-0.01
25	C	-0.01	-0.03	0.01
26	C	0.03	-0.01	0.01
28	C	0.01	-0.05	0.02
29	H	-0.09	0.02	-0.01
30	O	-0.01	0.00	0.00
31	C	0.05	-0.04	0.02
32	C	0.05	0.04	-0.02
33	H	-0.09	-0.02	0.01
34	C	0.01	0.05	-0.02
35	C	0.00	0.01	0.00
36	C	-0.07	0.12	-0.05
37	H	-0.14	-0.03	0.01
38	H	-0.14	0.03	-0.01
39	C	-0.07	-0.12	0.05
40	C	0.00	-0.01	0.00
41	H	0.00	-0.04	0.00
42	H	0.00	-0.03	0.03
43	H	0.02	0.01	0.00
44	N	0.09	-0.10	0.04
45	N	0.09	0.10	-0.04
46	H	0.00	0.03	0.00
47	H	0.02	-0.01	0.00
48	H	0.00	0.03	-0.03
49	C	-0.03	0.00	0.00
50	C	-0.03	0.00	0.00
51	H	-0.04	-0.05	-0.01
52	H	0.02	0.18	-0.08
53	C	0.00	0.01	0.00
54	H	0.02	-0.18	0.08
55	H	-0.04	0.05	0.01
56	C	0.00	-0.01	0.00
57	H	0.00	0.01	-0.01
58	H	0.03	0.01	0.00
59	H	-0.01	-0.04	-0.01
60	H	0.03	-0.01	0.00
61	H	0.00	-0.01	0.01
62	H	-0.01	0.04	0.01
63	H	-0.28	0.48	-0.19
64	H	-0.28	-0.48	0.19

Table S4. Atomic displacements of each atom involved in mode 149 in the unit of angstroms. The XYZ vectors are displayed in Fig. S13.

mode 150				
**atom number**	**element**	**X**	**Y**	**Z**
11	C	0.00	-0.02	0.00
12	C	0.00	0.01	0.01
13	C	0.00	0.01	0.00
14	C	0.00	0.02	-0.01
15	C	0.00	-0.01	0.00
16	H	0.00	0.01	0.01
17	C	0.00	-0.02	-0.01
18	C	-0.03	-0.08	0.03
19	C	0.03	-0.08	0.03
20	H	0.00	-0.01	0.00
21	C	0.00	0.02	0.01
22	H	0.00	0.01	0.03
23	C	0.07	0.01	-0.01
24	C	-0.04	0.11	-0.04
25	C	0.04	0.11	-0.04
26	C	-0.07	0.01	-0.01
27	H	0.00	-0.03	0.01
28	C	-0.06	-0.05	0.02
29	H	-0.08	0.02	-0.01
30	O	0.00	-0.02	0.01
31	C	0.00	-0.07	0.03
32	C	0.00	-0.07	0.03
33	H	0.08	0.02	-0.01
34	C	0.06	-0.05	0.02
35	C	0.01	0.01	-0.01
36	C	0.04	0.13	-0.06
37	H	-0.01	-0.08	0.03
38	H	0.01	-0.08	0.03
39	C	-0.04	0.13	-0.06
40	C	-0.01	0.01	-0.01
41	H	0.03	-0.06	-0.01
42	H	0.03	-0.05	0.06
43	H	-0.04	0.01	0.01
44	N	0.05	-0.09	0.03
45	N	-0.05	-0.09	0.03
46	H	-0.04	-0.06	-0.01
47	H	0.04	0.01	0.01
48	H	-0.03	-0.05	0.06
49	C	-0.02	0.01	0.00
50	C	0.02	0.01	0.00
51	H	0.00	-0.06	0.01
52	H	0.00	0.09	-0.03
54	H	0.00	0.09	-0.03
55	H	0.00	-0.06	0.01
57	H	0.00	0.01	0.00
58	H	0.02	0.00	-0.01
59	H	0.00	-0.03	0.00
60	H	-0.02	0.00	-0.01
61	H	0.00	0.01	0.00
62	H	0.00	-0.03	0.00
63	H	-0.32	0.49	-0.20
64	H	0.32	0.49	-0.20

Table S5. Atomic displacements of each atom involved in mode 150 in the unit of angstroms. The XYZ vectors are displayed in Fig. S13.

(d). Analysis of mode 153 (xanthene ring mode)

The calculated vibrational mode 153 in water at ~1650 cm^{-1} is local to the xanthene ring. The atoms involved and the displacements of each atom are shown in Fig. S16 and Table S6.

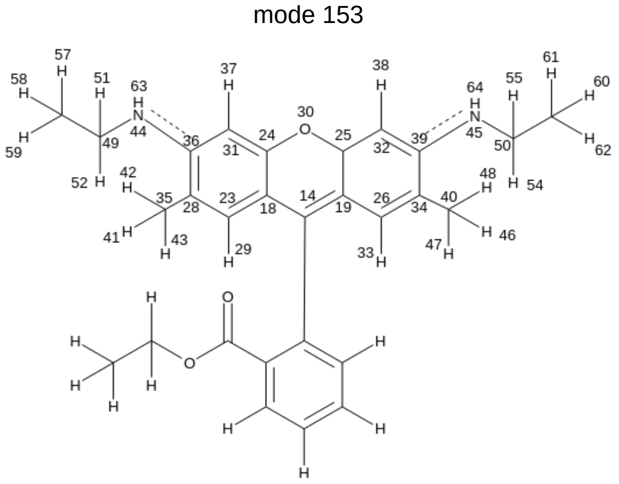

Fig. S16. Atoms involved in mode 153.

mode 153				
**atom number**	**element**	**X**	**Y**	**Z**
14	C	-0.07	0.00	0.00
18	C	-0.04	-0.01	0.00
19	C	-0.04	0.01	0.00
23	C	0.25	-0.07	0.03
24	C	0.10	-0.05	0.02
25	C	0.10	0.05	-0.02
26	C	0.25	0.07	-0.03
28	C	-0.25	0.08	-0.03
29	H	-0.34	-0.05	0.02
30	O	-0.01	0.00	0.00
31	C	-0.17	0.02	-0.01
32	C	-0.17	-0.02	0.01
33	H	-0.34	0.05	-0.02
34	C	-0.25	-0.08	0.03
35	C	0.03	0.01	0.00
36	C	0.13	0.03	-0.02
37	H	0.21	0.00	0.00
38	H	0.21	0.00	0.00
39	C	0.13	-0.03	0.02
40	C	0.03	-0.01	0.00
41	H	0.13	-0.07	-0.06
42	H	0.13	-0.02	0.10
43	H	-0.15	-0.01	0.01
44	N	0.00	-0.04	0.02
45	N	0.00	0.04	-0.02
46	H	0.13	0.07	0.06
47	H	-0.15	0.01	-0.01
48	H	0.13	0.02	-0.10
49	C	0.00	0.02	-0.01
50	C	0.00	-0.02	0.01
51	H	0.03	-0.06	0.01
52	H	-0.03	-0.04	0.02
54	H	-0.03	0.04	-0.02
55	H	0.03	0.06	-0.01
57	H	0.00	-0.01	0.01
58	H	-0.01	-0.02	-0.04
59	H	0.01	0.00	0.01
60	H	-0.01	0.02	0.04
61	H	0.00	0.01	-0.01
62	H	0.01	0.00	-0.01
63	H	-0.12	0.16	-0.07
64	H	-0.12	-0.16	0.07

Table S6. Atomic displacements of each atom involved in mode 153 in the unit of angstroms. The XYZ vectors are displayed in Fig. S13.

(e). Analysis of mode 155 (ester group mode)

The calculated vibrational mode 155 in water at ~1720 cm^{-1} is largely a local mode of the ester group. However, calculation results also reveal coupling to the atoms in the xanthene ring. This coupling could be the origin of IR-induced fluorescence intensity change of R6G when the IR laser frequency is tuned to 1720 cm^{-1} as discussed in the main text. The atoms involved and the displacements of each atom are shown in Fig. S17 and Table S7.

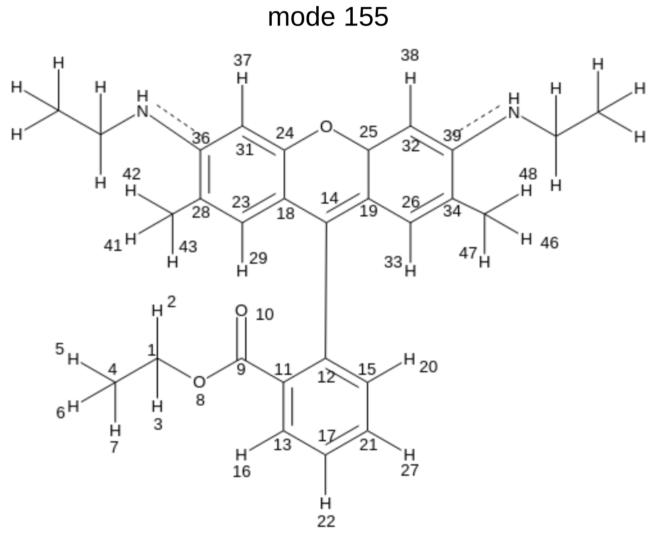

Fig. S17. Atoms involved in mode 155.

mode 155				
**atom number**	**element**	**X**	**Y**	**Z**
1	C	0.00	0.00	-0.04
2	H	-0.06	0.07	0.13
3	H	0.06	0.07	0.13
4	C	0.00	-0.01	0.01
5	H	0.01	-0.03	0.00
6	H	0.00	0.08	0.00
7	H	-0.01	-0.03	0.00
8	O	0.00	-0.05	0.03
9	C	0.00	0.64	-0.48
10	O	0.00	-0.41	0.29
11	C	0.00	-0.11	0.07
12	C	0.00	0.06	0.00
13	C	0.00	0.03	0.01
14	C	0.00	-0.02	0.00
15	C	0.00	-0.02	0.00
16	H	0.00	0.03	0.05
17	C	0.00	-0.03	-0.04
18	C	-0.02	0.01	0.00
19	C	0.02	0.01	0.00
20	H	0.00	0.01	-0.05
21	C	0.00	0.03	0.01
22	H	0.00	0.02	0.05
23	C	0.02	-0.01	0.00
24	C	0.02	-0.02	0.01
25	C	-0.02	-0.02	0.01
26	C	-0.02	-0.01	0.00
27	H	0.00	-0.03	0.01
28	C	-0.02	0.01	0.00
29	H	-0.04	0.00	0.00
31	C	-0.02	0.01	0.00
32	C	0.02	0.01	0.00
33	H	0.04	0.00	0.00
34	C	0.02	0.01	0.00
36	C	0.01	0.00	0.00
37	H	0.02	0.00	0.00
38	H	-0.02	0.00	0.00
39	C	-0.01	0.00	0.00
41	H	0.01	0.00	0.00
42	H	0.01	0.00	0.00
43	H	-0.01	0.00	0.00
46	H	-0.01	0.00	0.00
47	H	0.01	0.00	0.00
48	H	-0.01	0.00	0.00

Table S7. Atomic displacements of each atom involved in mode 155 in the unit of angstroms. The XYZ vectors are displayed in Fig. S13.

VI. Determination of scale bars, field of view, and spatial resolution of the MD-WISE images

(a). Determination of scale bars

The 3-micron silica spheres stained with fluorescent dyes (806765, Sigma Aldrich, see Section I. Materials and staining methods) were used as the calibration target for the MD-WISE microscope. The scale bars in all the MD-WISE fluorescent images were calculated based on the diameter of the images of the silica beads acquired under the same imaging conditions. The absolute size of the silica beads was in turn calibrated with a standard reticle (Thorlabs, R1L3S6PR) using an optical microscope equipped with a x100 objective under whitelight trans-illumination configuration. As shown below in Fig. S18, the diameter of the silica beads is 3 microns.

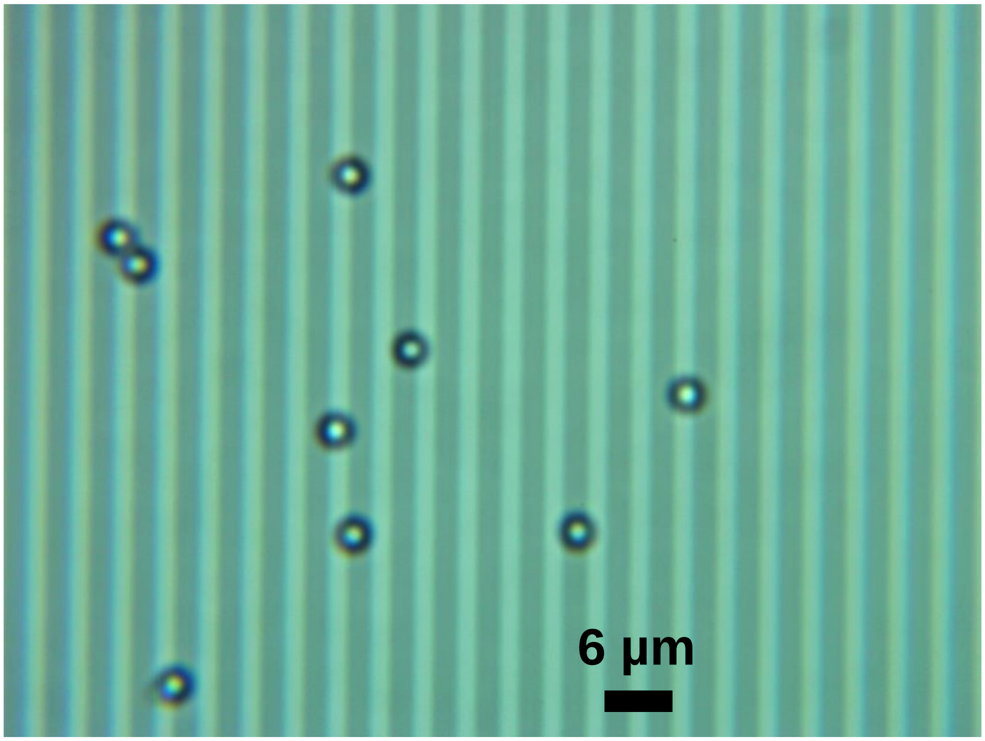

Fig. S18. The reticle has 250 line pairs (one light line and one dark line) per millimeter. Each light or dark line has a width of 2 microns. We can determine the diameter of the silica beads is 3 microns.

(b). An example of the full field of view.

For MD-WISE imaging experiments, the field of view is adjustable depending on the size of the objects that need to be imaged. For individual silica beads, we typically chose the field of view of 10~20 microns by setting the beam size of the IR and visible pulses to a value in this range. Such beam size is more than sufficient to image the 3-micron beads. To capture the fluorescence image of the entire field, we imaged a sample with crowded beads. As shown below in Fig. S19, the image of beads reports a field of view of ~15 microns. The variations of image intensity could be due to the inhomogeneous loading of dyes in the beads. For images of single cells, they were acquired with larger field of view, and the large size of the cells themselves (20-30 microns) nearly filled the entire field of view.

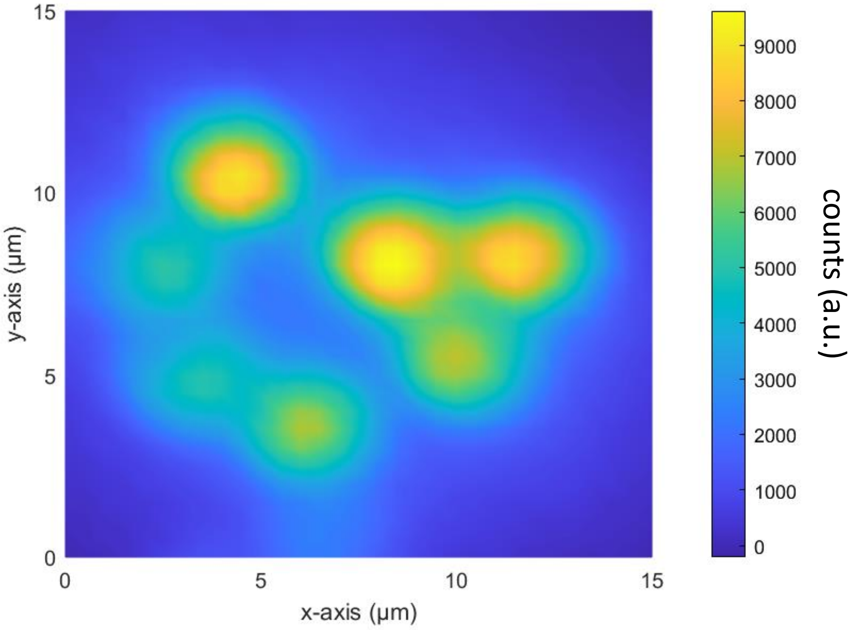

Fig. S19. Fluorescence image of 3-micron silica beads showing an entire field of view of ~15 microns.

(c). Determination of the spatial resolution.

First, we determined whether MD-WISE has a resolution as good as regular photoluminescence (PL) images in our setup. As shown by the linecuts of MD-WISE and PL images in Fig. S20, they lay on top of each other, with identical widths for both the 2-micron (left) and 3-micron (right) silica microspheres, respectively. This suggested that MD-WISE has as good resolution as regular PL imaging using either quantum dots or R6G dye as the chromophore. This makes sense, because the resolution of MD-WISE imaging is defined by the optical objective we used to resolve PL or fluorescence signals.

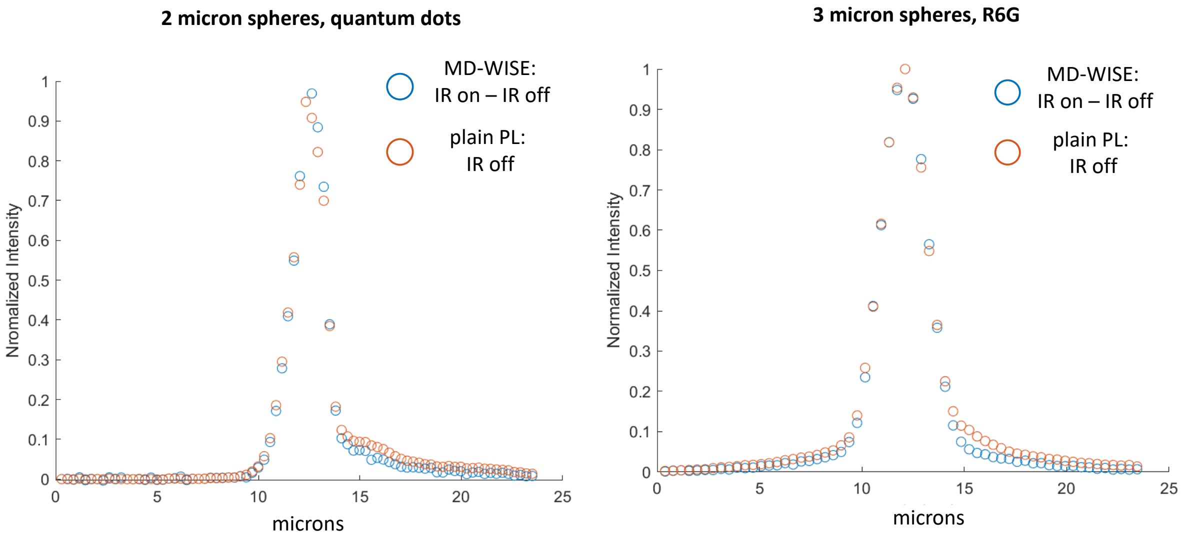

Fig. S20. Linecuts of MD-WISE (blue) and plain PL images (orange) acquired without IR modulation. The linecuts were performed across the images of individual silica spheres stained with either quantum dots or R6G dyes as shown in the main texts.

We then determined the resolution of our setup by analyzing the point spread function in regular PL images of individual perovskite nanocrystals (size 50 nm, composition $MAPbBr_3$) prepared according a literature method [7]. The nanocrystal serves as a bright PL point source of which the physical size is much smaller than the resolution limit. The FWHM of the gaussian fitting of the point spread function as shown in Fig. S21 yields a resolution of 1.6 microns.

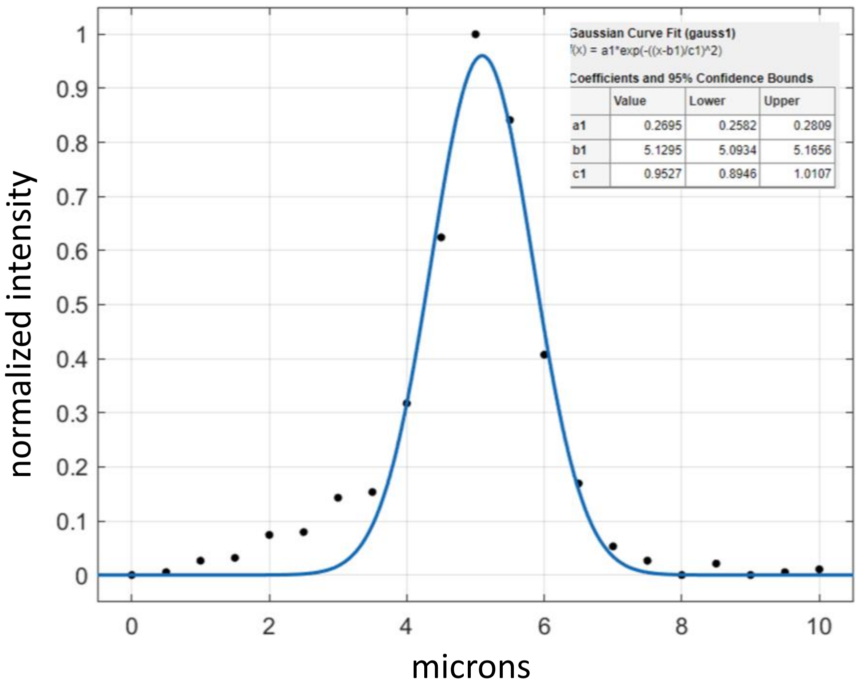

Fig. S21. Gaussian fitting of the point spread function measured using a bright nanocrystal. The fitting equation and results are embedded in the upright corner. The FWHM of the gaussian is 2.355 * (c1/1.414) = 1.6 microns.

SI References

1. B. Wu, J. P. Breen, X. Xing, M. D. Fayer, Controlling the Dynamics of Ionic Liquid Thin Films via Multilayer Surface Functionalization. *J Am Chem Soc* **142**, 9482–9492 (2020).
2. Gaussian 09, Revision D.01, M. J. Frisch, G. W. Trucks, H. B. Schlegel, G. E. Scuseria, M. A. Robb, J. R. Cheeseman, G. Scalmani, V. Barone, G. A. Petersson, H. Nakatsuji, X. Li, M. Caricato, A. Marenich, J. Bloino, B. G. Janesko, R. Gomperts, B. Mennucci, H. P. Hratchian, J. V. Ortiz, A. F. Izmaylov, J. L. Sonnenberg, D. Williams-Young, F. Ding, F. Lipparini, F. Egidi, J. Goings, B. Peng, A. Petrone, T. Henderson, D. Ranasinghe, V. G. Zakrzewski, J. Gao, N. Rega, G. Zheng, W. Liang, M. Hada, M. Ehara, K. Toyota, R. Fukuda, J. Hasegawa, M. Ishida, T. Nakajima, Y. Honda, O. Kitao, H. Nakai, T. Vreven, K. Throssell, J. A. Montgomery, Jr., J. E. Peralta, F. Ogliaro, M. Bearpark, J. J. Heyd, E. Brothers, K. N. Kudin, V. N. Staroverov, T. Keith, R. Kobayashi, J. Normand, K. Raghavachari, A. Rendell, J. C. Burant, S. S. Iyengar, J. Tomasi, M. Cossi, J. M. Millam, M. Klene, C. Adamo, R. Cammi, J. W. Ochterski, R. L. Martin, K. Morokuma, O. Farkas, J. B. Foresman, and D. J. Fox, Gaussian, Inc., Wallingford CT, 2016.
3. W. J. Hehre, R. Ditchfield, J. A. Pople, Self-Consistent Molecular Orbital Methods. XII. Further Extensions of Gaussian—Type Basis Sets for Use in Molecular Orbital Studies of Organic Molecules. *J Chem Phys* **56**, 2257–2261 (1972).
4. C. Lee, W. Yang, R. G. Parr, Development of the Colle-Salvetti correlation-energy formula into a functional of the electron density. *Phys Rev B* **37**, 785–789 (1988).
5. D. N. Adhikesavalu, D. Mastropaolo, A. Camerman, N. Camerman, Two rhodamine derivatives: 9-[2-(ethoxycarbonyl)phenyl]-3,6-bis(ethylamino)-2,7-dimethylxanthylium chloride monohydrate and 3,6-diamino-9-[2-(methoxycarbonyl)phenyl]xanthylium chloride trihydrate. *Acta Crystallographica C* **57**, 657–659 (2001).
6. H. Watanabe, N. Hayazawa, Y. Inouye, S. Kawata, DFT Vibrational Calculations of Rhodamine 6G Adsorbed on Silver: Analysis of Tip-Enhanced Raman Spectroscopy. *J Phys Chem B* **109**, 5012–5020 (2005).
7. T. Tachikawa, I. Karimata, Y. Kobori, Surface Charge Trapping in Organolead Halide Perovskites Explored by Single-Particle Photoluminescence Imaging. *J Phys Chem Lett* **6**, 3195-3201 (2015).
